# Proteomic and transcriptomic studies of HBV-associated liver fibrosis of an AAV-HBV-infected mouse model

**DOI:** 10.1186/s12864-017-3984-z

**Published:** 2017-08-22

**Authors:** Fangming Kan, Lei Ye, Tao Yan, Jiaqi Cao, Jianhua Zheng, Wuping Li

**Affiliations:** MOH Key Laboratory of Systems Biology of Pathogens, Institute of Pathogen Biology, Chinese Academy of Medical Sciences & Peking Union Medical College, Beijing, China

**Keywords:** Liver fibrosis, HBV, Proteomics, Transcriptomics

## Abstract

**Background:**

Human hepatitis B virus (HBV) infection is an important public health issue in the Asia-Pacific region and is associated with chronic hepatitis, liver fibrosis, cirrhosis and even liver cancer. However, the underlying mechanisms of HBV-associated liver fibrosis remain incompletely understood.

**Results:**

In the present study, proteomic and transcriptomic approaches as well as biological network analyses were performed to investigate the differentially expressed molecular signature and key regulatory networks that were associated with HBV-mediated liver fibrosis. RNA sequencing and 2DE-MALDI-TOF/TOF were performed on liver tissue samples obtained from HBV-infected C57BL/6 mouse generated via AAV8-HBV virus. The results showed that 322 genes and 173 proteins were differentially expressed, and 28 HBV-specific proteins were identified by comprehensive proteomic and transcriptomic analysis. GO analysis indicated that the differentially expressed proteins were predominantly involved in oxidative stress, which plays a key role in HBV-related liver fibrosis. Importantly, CAT, PRDX1, GSTP1, NXN and BLVRB were shown to be associated with oxidative stress among the differentially expressed proteins. The most striking results were validated by Western blot and RT-qPCR. The RIG-I like receptor signaling pathway was found to be the major signal pathway that changed during HBV-related fibrosis.

**Conclusions:**

This study provides novel insights into HBV-associated liver fibrosis and reveals the significant role of oxidative stress in liver fibrosis. Furthermore, CAT, BLVRB, NXN, PRDX1, and IDH1 may be candidates for detection of liver fibrosis or therapeutic targets for the treatment of liver fibrosis.

**Electronic supplementary material:**

The online version of this article (doi:10.1186/s12864-017-3984-z) contains supplementary material, which is available to authorized users.

## Background

The human hepatitis B virus (HBV), which belongs to the *Hepadnaviridae* family*,* is a small, enveloped, partially double-stranded DNA virus. In the biological host, the virus can cause acute and chronic hepatitis. The key characteristic of the HBV virus is its high species and cell-type specificity. Although an effective vaccine and 7 therapeutics (2 immunomodulatory agents and 5 nucleotide analogs) are available, chronic HBV infections still pose a major threat to humans and are a severe public health burden worldwide. Approximately 400 million people globally are chronically infected, especially those who are at high risk for liver fibrosis, cirrhosis, and hepatocellular carcinoma (HCC) [1–3]. Liver fibrosis, which has various causes, is a common pathological process of liver injury characterized by excessive accumulation of extracellular matrix. It is an important health issue that affects 100 million people in many parts of the world and has high morbidity and mortality rates [4].

Chronic hepatitis B virus infection remains the major cause of liver injury and fibrosis. When liver damage occurs, the inflammatory response is stimulated to secrete fibrogenic cytokines, such as TGF-β1, angiotensin II, and leptin. These responses activate collagen-producing cells, including hepatic stellate cells (HSCs), portal fibroblasts, and myofibroblasts. The activated collagen-producing cells synthesize collagen, which changes the quantity and composition of extracellular matrix (ECM) [5]. When the liver injury persists, damaged hepatocytes are substituted with fibrillar collagen and distributed around portal tracts and/or pericentral areas [6]. As the liver injury progresses, the extent of liver fibrosis can progress from collagen bands to bridging fibrosis and cirrhosis.

Although major progress has been made in elucidating the mechanisms of viral hepatitis and its complications, there are still many clinical problems that are not fully understood and crucial questions that remain unanswered. In chronic HBV infections, the role of the immune system and the molecular mechanisms of liver fibrosis are still unclear. The reason for these shortcomings is largely due to the lack of reliable in vivo infection systems and most importantly the difficulty in obtaining convenient small animal models. Currently, three types of small animal models with liver fibrosis are commonly used (HBV transgenic mice, carbon tetrachloride-induced rat model, and bile duct ligation rat model) [7–9]. The HBV transgenic mouse provides a reliable HBV replication model to study the molecular mechanisms of liver diseases. However, the viral genome, which is integrated into the host genome and the immune system, recognizes the virus as itself. The HBV genome cannot be eliminated from the liver cells of the mouse because its use is limited to research purposes, antiviral drug screening, and evaluation. The other two animal models of liver fibrosis do not mimic the liver fibrosis elicited by HBV chronic infection.

Proteomics and transcriptomics have emerged as promising techniques in analyzing comprehensive protein and gene expression profiles [10, 11]; these analyses can elucidate complex pathogenic mechanisms [12, 13]. Major progress has been made in the elucidation of pathogenesis, the identification of biomarkers and the staging of fibrosis using proteomic approaches in HBV-related fibrosis [14, 15]. By studying serum samples at different stages of fibrosis, Jia [16] and Karatayli [17] identified several non-invasive markers. Peroxiredoxin 2 [18], Annexin A2 [19] and several other proteins were also shown to be biomarkers. Additionally, several liver fibrosis indexes were established, for example, the ANN fibrosis indices [20]. Although serum is an important source of material for studying liver fibrosis and identifying biomarkers, liver tissue, which interacts directly with the virus, should not be neglected. The study of liver tissue at different stages of fibrosis will help to reveal the mechanism of virus-host interactions and fibrosis. Additionally, the mechanism of hepatic fibrosis remains unclear. Because HBV-induced liver fibrosis is a dynamic and complex process, a single omics study may not adequately reflect the changes in this disease. Thus, integrated omics approaches are essential, and in the present study, multiple omics analyses of this complex liver disease were performed. Profiling the proteomic and transcriptomic changes in HBV infection, especially HBV-induced liver fibrosis, is pivotal to elucidate the mechanisms of HBV-associated diseases. There are several proteomic studies of liver fibrosis, but these results came from the liver tissue of an HBV transgenic mouse [21, 22]. In contrast, we recently developed an HBV persistent infection mouse model using AAV-8-mediated hepatic delivery of the HBV genome [23]. This mouse line exhibited chronic HBV replication with viral antigen production and viremia lasting more than 6 months, which was accompanied by liver fibrosis, and recapitulates the reaction that occurs in humans during persistent HBV infection.

In the present study, a mouse model of persistent HBV infection and liver fibrosis was used to investigate the HBV-specific proteomic and transcriptomic signature using RNA sequencing (RNA-Seq) and 2D–MALDI-TOF/TOF. A total of 213 differentially expressed genes and 173 proteins were identified, respectively. Twenty-eight altered proteins were found by comprehensive proteomic and transcriptomic analyses. The hepatic stellate cells play multiple crucial roles in the physiology and pathology of the liver. Activated HSCs are the major source of the extracellular matrix and cytokines, which further regulate the process of liver fibrosis [24]. Therefore, we analyzed several differentially expressed proteins in LX2 (HSC cell line) to validate these changes in vivo. Bioinformatics analysis suggested that proteins involved in oxidative stress may play a key role in the development of liver fibrosis. Additionally, a RIG-I-like receptor signaling pathway was found to be the major signal pathway that changed during HBV-related fibrosis. This study identified key gene transcription and expression profiles for diagnosis and therapeutic targets of liver fibrosis and may provide further insight into the mechanism of liver fibrosis.

## Results

### The establishment and characteristics of the liver fibrosis mouse model

**Fig. 1:**
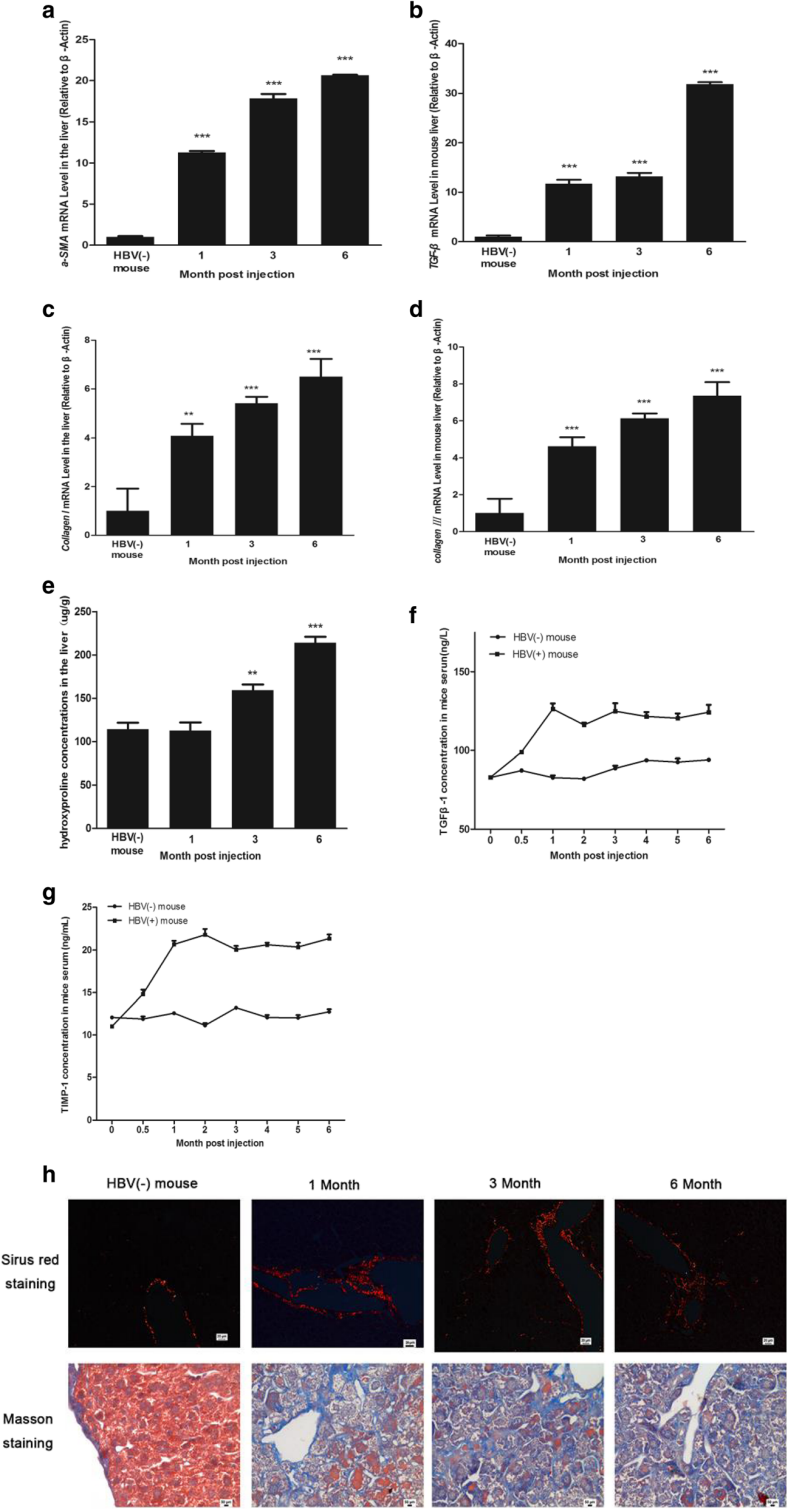
Establishment of a mouse model with persistent HBV infection and liver fibrosis. The mice were injected with AAV-HBV vector (2 × 10^11^ viral genome equivalents (vg)) via the tail vein. The control mice were injected with the same volume of PBS. Then, serum was collected at 1, 3 and 6-months post injection. The parameters associated with persistent infection and liver fibrosis were analyzed. **a**–**d** The expression levels of α-SMA, TGF-β, Collagen I and Collagen III were assessed using quantitative RT-PCR in the liver tissue of HBV (−) and HBV (+) mice. **e**–**g** The concentration of Hydroxyproline, TGF-β and TIMP1 in the serum was assessed by ELISA. **h** The liver tissues were collected at 1, 3 and 6-montha post injection. Masson’s stain and Sirus red staining were performed to assess collagen deposition. Significantly increased fibrosis was found in the HBV (+) mouse. Representative images of Sirius red (*upper panels*) and Masson’s staining (*lower panels*) of liver sections at the indicated time points are shown. Statistical analyses were performed using a one-way analysis of variance. The data represent the mean ± SD (*n* = 4). Compared to HBV (−) mouse:* *P* < 0.05, ** *P* < 0.01,*** *P* < 0.001

Our previous study demonstrated that AAV-HBV injection can induce liver fibrosis [23]. To construct the mouse model of liver fibrosis, AAV-HBV1.2 at 2 + e^11^ vg was injected into the tail vein of a live mouse. Liver tissue and serum were collected at different time points after injection. To characterize liver fibrosis, the mRNA and protein levels of a number of fibrogenic biomarkers and extracellular matrix (ECM) proteins were examined. The biomarkers and proteins clearly showed that the livers from the mice treated with AAV-HBV became fibrotic. RT-qPCR examination of α-SMA (Fig. 1a), TGF-β (Fig. 1b), collagen I (Fig. 1c), and collagen III (Fig. 1d) showed that they were significantly up-regulated in the liver of the mouse model compared to the control. ELISA detection of Hydroxyproline (Fig. 1e), TGF-β (Fig. 1f) and TIMP-1 (Fig. 1g) concentration in the serum also confirmed that these proteins showed a steady increasing trend during the 6-month period. These proteins are well-established biomarkers of liver fibrosis. To examine the morphological changes of liver tissues after AAV-HBV treatment, Masson’s trichrome staining and Sirius red staining were performed to detect collagen deposition and fiber production. These tests clearly showed fibrogenesis in the liver of the mouse model. As indicated in Fig. 1h, an increasing trend of extensive collagen deposition and fiber production was observed in the liver tissues of the AAV-HBV treated mouse during the 6-month period. Fibrous expansion around the portal areas with marked bridging was observed in the liver of the mouse model. By contrast, the liver of the untreated mouse exhibited normal morphology.

### Proteomic identification of proteins associated with liver fibrosis and bioinformatics analysis

**Fig. 2:**
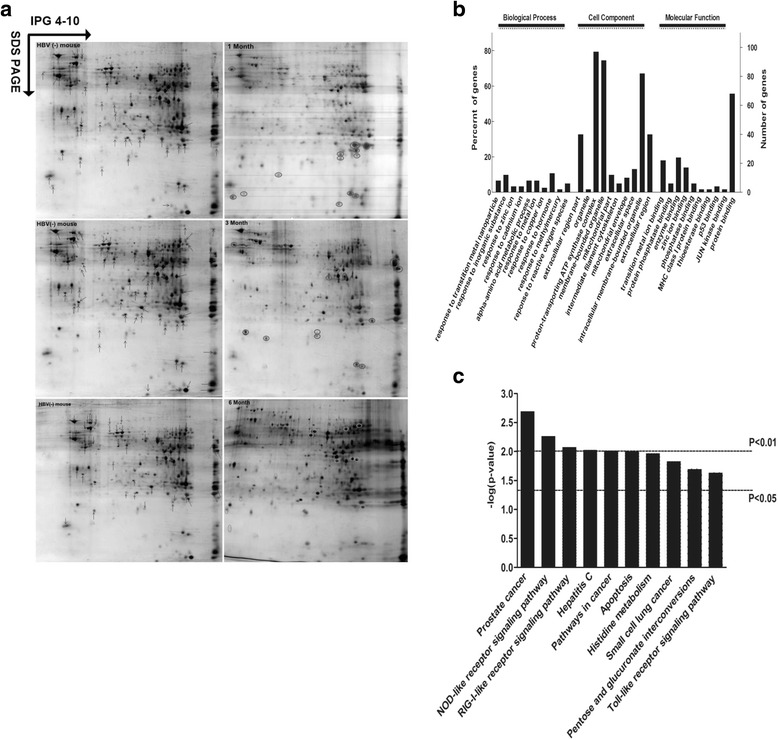
Proteomic analyses of the HBV mouse model. **a** Two-dimensional gel electrophoresis analysis of the liver tissue in HBV (+) mice and HBV (−) mice. The liver tissues of HBV (+) and HBV (−) mice were collected at 1, 3 and 6-month post-injection. Total proteins were extracted, and 300 μg protein was analyzed by two-dimensional gel electrophoresis (pH 3–10 NL). The representative pictures of 2-D gel images are shown: the left panels are the 2-D gels of HBV (−) mice, and the right panels are the 2-D gels of HBV (+) mice. Arrows represent the down-regulated proteins, and the circle represents the up-regulated proteins compared with the HBV (−) mice. The experiments were performed in triplicate. **b** GO analysis of the differently expressed proteins. The chart shows an overview of the gene ontology analysis with up to 10 significantly enriched terms in the BP, CC, and MF categories. The cut-off of *P*-value is set to 0.05. Terms of the same category are ordered by *P*-values. Left terms are more significant than the right. Information on the percentage and number of involved proteins in a term is shown on the y-axis. **c** KEGG Pathway analysis of the differentially expressed proteins. The chart shows the top 10 enriched KEGG Pathways. *P* value =0.01 and *P* value =0.05 as two selected cutoffs are highlighted on the figure

Proteins are the functional molecules of cells. To gain a better understanding of the changes of HBV-related fibrosis at the protein level, the liver tissues from the HBV (−) and HBV (+) mice were assessed using 2-DE and MALDI-TOF/TOF MS during the 6-month period. These samples were analyzed by two-dimensional gel electrophoresis in triplicate. The representative 2-DE maps with immobilized pH gradient (IPG) strips of pH 4–10 are shown in Fig. 2a. Analysis using Image Master showed that more than 1500 protein spots of each gel were detected with good reproducibility in the mass range of 15–120 kDa. Of the 1500 proteins, more than 173 differentially accumulated protein spots, which showed a more than 1.5-fold or less than 0.66-fold change [25] and showed the difference in at least two parallel gels, were successfully identified. Compared to the HBV (−) mice, 79 (46%) of the differentially expressed proteins were up-regulated, whereas 94 (54%) were down-regulated. Notably, among these proteins, glutathione S-transferase P 2 (GSTP2) showed differential expression throughout the 6-month period.

As shown by GO (Fig. 2b) and KEGG (Fig. 2c) analysis, the differentially expressed proteins were predominantly clustered in response to metal ions, reactive oxygen species, MHC class I protein binding, enzyme binding, the component of proton-transporting ATP synthase complex, and mitochondrial part. KEGG analysis indicated that the identified proteins were enriched in 19 pathways (count > 2, *p* < 0.05), including the NOD-like receptor signaling pathway, the RIG-I-like receptor signaling pathway, apoptosis, and the Toll-like receptor signaling pathway, which are related to the immune system. To further understand the functional associations of the identified proteins, a protein-protein interaction (PPI) network was generated using STRING (Additional file 1: Fig. S1A) and Cytoscape software (Additional file 1: Fig. S1B). The results indicated that the interacting proteins have important functions in the clusters of amino acid metabolism, peroxisome, B cell receptor signaling pathway, and cancer and T cell receptor signaling pathway. These results suggest that the differentially expressed proteins may play an important role in liver fibrosis by participating in liver metabolism, immune regulation, and oxidative stress response.

### Analysis of RNA-Seq data

**Fig. 3:**
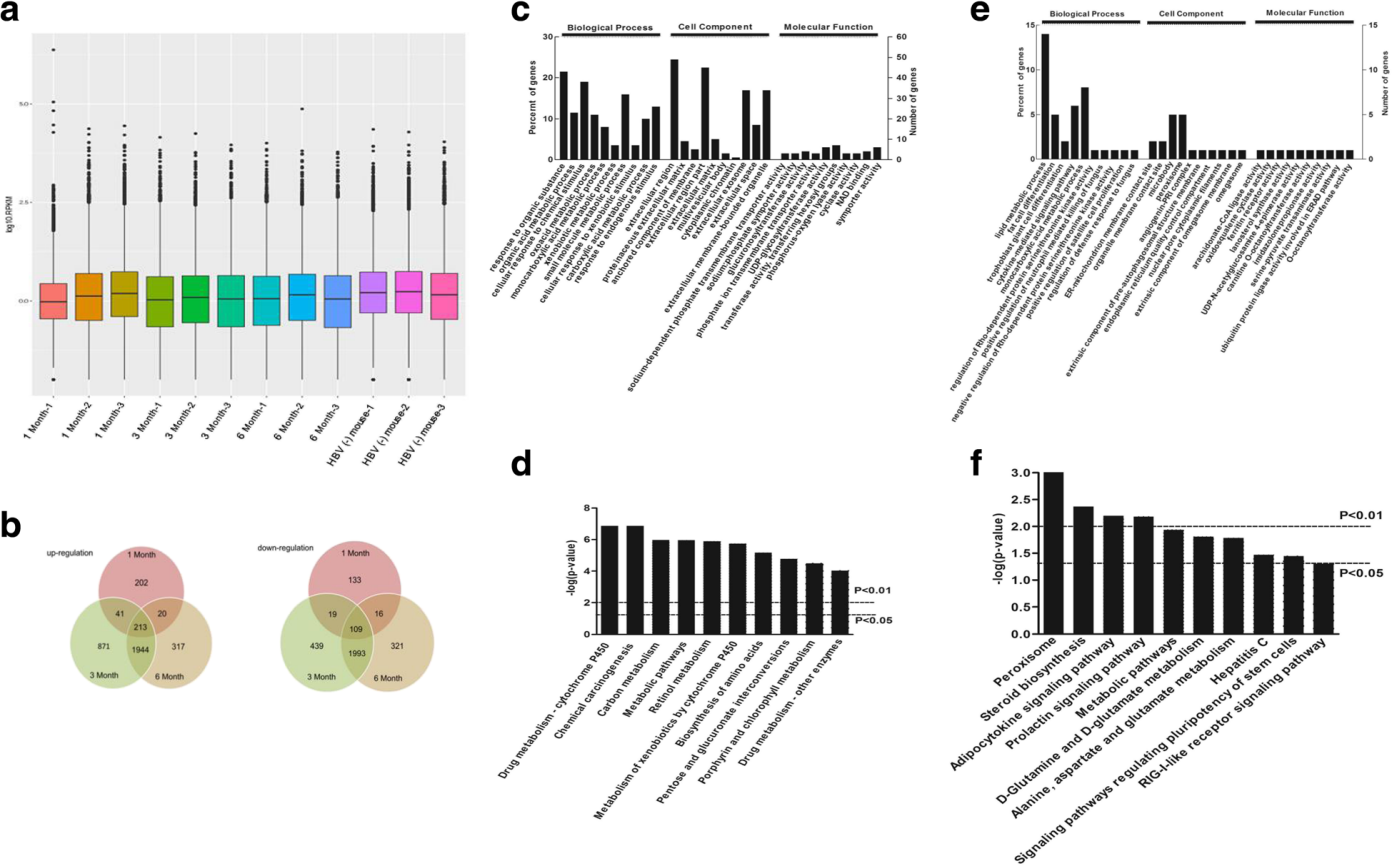
Transcriptomic analysis of the HBV mouse model. The total RNA of liver tissue in the HBV (+) mouse and HBV (−) mouse were extracted at 1, 3 and 6-months post injection. Then, RNA-Seq analysis was performed to assess the differentially expressed genes. The experiments were performed in triplicate. **a** RPKM box plot shows the distribution of gene expression in different samples. The abscissa is the sample name, and the ordinate is log10 (RPKM). Each region of the box icon notes five statistics: the maximum value, Q3 quartile, the median, Q1 quartile, and the minimum value. **b** Venn diagram of up-regulated and down-regulated genes from the HBV (+) mouse liver tissues at 1, 3 and 6 months post-injection. **c**, **e** GO analysis of the differently expressed proteins. The up-regulated (**c**) and down-regulated (**e**) genes in the HBV (+) mouse were analyzed by Gene Ontology. The charts show an overview of the gene ontology analysis with up to 10 significantly enriched terms in the BP, CC, and MF categories. The cut-off of the *P*-value is set to 0.05. Terms of the same category are ordered by *P*-values. Left terms are more significant than the right terms. Information about the percentage and number of involved genes in a term is shown at the y-axis. The KEGG Pathway analysis of the differentially expressed proteins. The up-regulated (**d**) and down-regulated (**f**) genes in the HBV (+) mouse were analyzed by KEGG Pathway. The top 10 enriched processes are shown here. *P* value =0.01 and *P* value =0.05 as two selected cutoffs are highlighted in the figure

To better understand the differentially transcribed genes at the RNA level, four cDNA libraries in triplicate of different groups ranging from 1, 3, and 6-months post injection were constructed and analyzed using RNA-Seq. Sequencing of the clean reads resulted in 29–40 million reads per sample, for a total of approximately 438 million reads across 12 samples. More importantly, all samples had 91–95% of the total reads that mapped to the mouse genome. Specifically, an average of 16,782; 17,088; 18,701; and 19,364 genes were identified (the reads per kilobase of transcript per megabase library size (RPKM) > 0.1) in the HBV (+) mouse (1, 3, 6-months post injection) and HBV (−) mouse (control groups), respectively. The mean RPKM value across all samples was 16.29, and the mean RPKM was 25.61 for genes with a RPKM > 1. Additionally, the distribution of the average RPKM per transcript was consistent between the four libraries (Fig. 3a). These results demonstrate a high degree of coverage, a common uniformity in transcriptomic composition, and a lack of overall shifts in transcript levels across samples. Therefore, it is probable that any differences seen in the subsequent analysis are the result of time point effects, not the result of sequencing or sample bias.

The differentially expressed genes (DEGs) were defined as genes with an adjusted *p*-value < 0.05 after FDR correction. Compared with the HBV (−) mouse, 753, 5629, and 4933 genes were significantly differentially transcribed in the HBV (+) mouse at every time point. In total, 6638 genes were found to be differentially expressed, of which 3608 were up-regulated and 3030 were down-regulated. Among these differentially expressed genes, 213 and 109 genes were up-regulated or down-regulated in all three HBV (+) mouse libraries (Fig. 3b).

To elucidate the roles and functions of these differentially expressed genes, GO analysis was performed to analyze the intersecting up-regulated (Fig. 3c) and down-regulated (Fig. 3e) genes. As observed in GO analysis, for the up-regulated genes, the highly enriched GO terms are associated with organic substance, organic acid metabolic process, sodium-dependent phosphate transmembrane transporter activity, NAD binding, extracellular region, proteinaceous extracellular matrix, and an anchored component of the membrane. The highly enriched GO terms in the down-regulated genes are associated with lipid metabolic process, fat cell differentiation, cytokine-mediated signaling pathway, negative regulation of Rho-dependent protein serine/threonine kinase activity, arachidonate-CoA ligase activity, ER-mitochondrion membrane contact site, organelle membrane contact site, and peroxisome.

KEGG results showed that these 213 up-regulated genes and 109 down-regulated genes mapped to 123 and 102 pathways, respectively. The 10 highly enriched pathways are shown in Fig. 3d and f. Drug metabolism-cytochrome P450, chemical carcinogenesis, peroxisome, steroid biosynthesis, and adipocytokine signaling pathways are the most enriched pathways that are associated with HBV infection and liver fibrosis.

To further understand the functional associations of the identified proteins, a protein-protein interaction (PPI) network was generated using STRING (Additional file 1: Fig. S2A, D) and Cytoscape software (Additional file 1: Fig. S2B, C, E). The identified proteins have important functions in chemical carcinogenesis, drug metabolism-cytochrome, retinol metabolism, ECM-receptor interaction, PI3K-Akt signaling pathway, AMPK signaling pathway, metabolic pathways, and the Jak-STAT signaling pathway. Taken together, these results suggest that the differentially expressed proteins may affect the progression of liver fibrosis by contributing in liver metabolism and ECM-receptor interactions. More importantly, the PI3K-Akt, AMPK, and Jak-STAT signaling pathways, which are associated with cell proliferation, differentiation, apoptosis, and immune regulation, are activated during the process of fibrosis.

### The comparison of proteomic and transcriptomic data

**Fig. 4:**
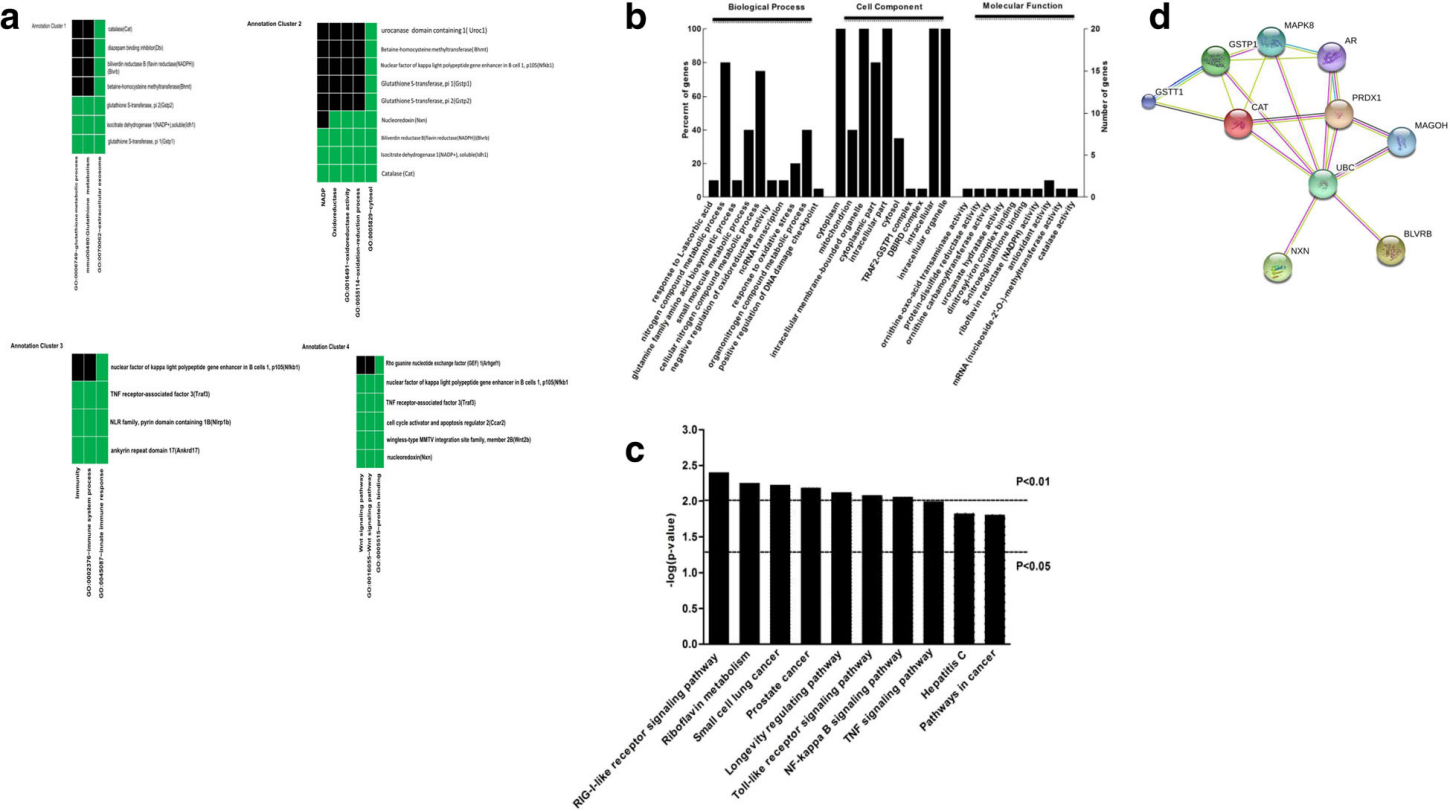
Integrated analyses of proteomics and transcriptomics. **a** Clustering analysis of the overlapped genes. DAVID analysis was used to analyze the 28 overlapped genes. Four enriched clusters are shown. *Green*: corresponding gene-term association positively reported; *Black*: corresponding gene-term association that was not yet reported. **b** GO analysis of the overlapping differently expressed proteins. Enriched Gene Ontology shows an overview of the gene ontology analysis with up to 10 significantly enriched terms in the BP, CC, and MF categories. The cut-off of *P*-value is set to 0.05. Terms of the same category are ordered by *P*-values. The left terms are more significant than the right terms. Information about the percentage and number of involved genes/proteins in a term is shown on the y-axis. **c** The KEGG Pathway analysis of the overlapping differentially expressed proteins. The overlapping differentially expressed proteins were analyzed using the KEGG Pathway. The top 10 enriched processes are shown here. *P* value =0.01 and *P* value =0.05 as two selected cutoffs are highlighted on the chart, as an indicator to show how significant the results are based on genome background enrichment. **d** The interaction analysis of proteins involved in oxidative stress, including GSTP1, CAT, PRDX1, NXN, and BLVRB, which is a key biological process during liver fibrosis. Further assessment of the five proteins was performed using STRING, and no more than five interactors were added

In this study, expression profiles of mRNAs were assessed using RNA-Seq analysis, while proteomic profiles were obtained using 2D–MALDI-MS/MS. Next, the differentially modulated proteins were compared to the differentially transcribed genes; 28 overlapped proteins were found (Table 1). Of these genes, 9 genes were localized to the cytoplasm, 8 to the nucleus, and 5 to the mitochondria. Additionally, most of the genes were differentially expressed at 3-months post injection. Then, we performed a cluster analysis of the overlapped genes through DAVID 6.7. As shown in Fig. 4a (Additional file 1: Table S1), the overlapped genes are in 4 clusters with an enrichment score > 1.0. Genes that were enriched in clusters with higher scores are involved in glutathione metabolic process, oxidation-reduction process, immune system process, and the Wnt signaling pathway.

**Table 1:** Transcriptomic and proteomic analysis of the overlapped genes and their corresponding proteins in HBV (+) mouse

		Fold Change							
		Transcriptomic			Proteomic			protein	
Gnen ID	Location	1 M	3 M	6 M	1 M	3 M	6 M	Function	Protein
ENSMUSG 00000038155	cytoplam;	4.70	8.8	8.27	N	up	N	dinitrosyl-iron complex binding; glutathione peroxidase activity; glutathione transferase activity; S-nitrosoglutathione binding	Gstp2
ENSMUSG 00000034456	cytosol	N	−2.55	−2.70	N	N	down	urocanatehydratase activity	Uroc1
ENSMUSG 00000074768	cytosol, extracellular	N	−8.56	−6.96	N	up	N	zinc ion binding, S-adenosylmethionine-homocysteine S-methyltransferase activity	Bhmt
ENSMUSG 00000039206	lysosome	N	−2.91	−2.65	N	N	up	metal ion binding, acylglycerol lipase activity	Daglb
ENSMUSG 00000026385	endoplasmic reticulum, extracellular	N	−3.00	−2.46	N	N	down	lipid binding	Dbi
ENSMUSG 00000033712	nuclear chromatin	N	−2.82	−3.30	N	N	down	RNA polymerase II core binding, enzyme inhibitor activity	Ccar2
ENSMUSG 00000032235	nucleus,	N	−2.46	−2.81	N	N	down	protein binding	Ice2
ENSMUSG 00000025950	cytosol, extracellular, peroxisome	N	−2.57	−2.05	N	N	down	isocitrate dehydrogenase (NADP+) activity	Idh1
ENSMUSG 00000031173	Mitochondrion	N	−3.42	−3.08	down	N	N	ornithine carbamoyltransferase activity	Otc
ENSMUSG 00000041268	extracellular	N	−2.80	−2.62	down	N	N	RabGTPase binding	Dmxl2
ENSMUSG 00000027187	cytosol, extracellular, peroxisome	N	−2.28	−2.00	N	down	N	antioxidant activity	Cat
ENSMUSG 00000046441	Nucleus. Cytoplasm.	N	−3.36	−3.90	N	N	down	mRNA (nucleoside-2-O-)-methyltransferase activity	Cmtr2
ENSMUSG 00000036591	cytoskeleton, golgi apparatus	N	−2.63	−3.54	N	N	down	GTPase activator activity	Arhgap21
ENSMUSG 00000021277	cytosol, mitochondrion	N	−2.81	−2.60	N	N	down	protein binding, tumor necrosis factor receptor binding	Traf3
ENSMUSG 00000030934	mitochondrion	N	−2.09	−3.03	N	down	N	ornithine-oxo-acid transaminase activity	Oat
ENSMUSG 00000055204	nucleus	N	4.41	3.712	N	up	up	chromatin binding	Ankrd17
ENSMUSG 00000071657	endoplasmic reticulum	N	2.88	3.53	N	N	up	protein binding	Bscl2
ENSMUSG 00000070390	cytosol, nucleus	N	8.16	5.02	N	N	up	protein binding	Nlrp1b
ENSMUSG 00000001674	nucleus	N	3.94	3.10	N	N	up	ATP binding, protein binding	Ddx18
ENSMUSG 00000040466	cytosol, extracellular, nucleus	N	2.35	3.03	N	N	up	biliverdinreductase activity, riboflavin reductase (NADPH) activity	Blvrb
ENSMUSG 00000020844	cytosol, nucleus	N	4.28	2.74	N	up	N	thioredoxin-disulfide reductase activity	Nxn
ENSMUSG 00000028163	cytosol, mitochondrion, nucleus	N	2.99	2.22	N	down	N	DNA binding	Nfkb1
ENSMUSG 00000024613	nucleus	N	6.15	5.93	N	up	N	skeletal system development	Tcof1
ENSMUSG 00000040940	cytosol,plasma membrane	N	2.87	2.24	N	N	down	protein binding, GTPase activator activity	Arhgef1
ENSMUSG 00000027840	extracellular	N	13.99	7.21	N	N	up	protein binding	wnt2b
ENSMUSG 00000055884	nucleus	5.70	N	−2.35	up	N	N	chromatin binding	Fancm
ENSMUSG 00000060803	Cytoplasm. Mitochondrion	N	N	−8.02	down	down	down	JUN kinase binding	Gstp1
ENSMUSG 00000041324	extracellular	N	N	4.30	N	up	N	cytokine activity	Inhba

N: no difference

up: up-regulated expression, compared with HBV(−) mouse

down: down-regulated expression, compared with HBV(−) mouse

GO and KEGG were used to analyze these overlapped genes, as shown by the GO (Fig. 4b) and KEGG (Fig. 4c) analyses. The 28 overlapped proteins were highly enriched in response to L-ascorbic acid, negative regulation of oxido-reductase activity, ncRNA transcription, response to oxidative stress, riboflavin reductase (NADPH) activity, and antioxidant activity. The RIG-I-like receptor signaling pathway is an overlapping pathway that was identified from proteomic analysis and RNA-Seq analysis. These bioinformatics results further suggest that redox reactions and innate immunity may play important roles in the process of liver fibrosis. Glutathione metabolism and redox reactions are closely related to the metabolism of intracellular reactive oxygen species, which is one of the major factors leading to liver fibrosis. Gstp1, Gstp2, Idh1, Blvrb, Cat, Idh1 and Nxn were shown to be involved in intracellular reactive oxygen species. In addition, SOD1 (down-regulated) and PRDX1 (up-regulated) were shown to be differentially expressed by proteomic analysis. However, liver fibrosis is the result of long-term interaction between the virus and host, and virus infection can disrupt the host immune system. Our results showed that four of the twenty-eight overlapped proteins, Nlrp1b, Ankrd17, traf3 and Nfkb1, are involved in the RIG-I-Like signaling pathway, which is associated with innate immunity. Oxidative stress has emerged as a central player in the development of liver fibrosis. The differentially expressed proteins CAT, PRDX1, GSTP1, NXN, and BLVRB are associated with oxidative stress. As observed in PPI analysis, we identified a key protein, Polyubiquitin-C (UBC), that could react with all five proteins (Fig. 4d).

### Validation of the changes in protein expression and gene transcription

**Fig. 5:**
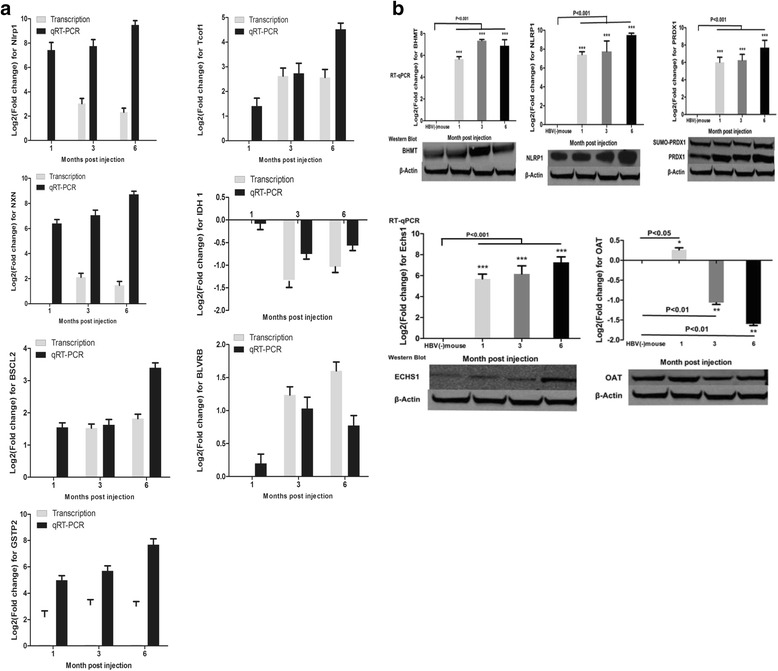
Verification of the selected differentially expressed genes by RT-qPCR and Western blot. **a** Log 2 fold change and RT-qPCR analysis of representative genes identified by RNA-seq. The expression of *Nlrp1, Tcof1, Idh1, Nxn, Blvrb and Bscl2* in the mouse liver tissue was verified by RT-qPCR. **b** Western blot and RT-qPCR analysis of representative proteins identified by 2D–MALDI-TOF/TOF. The expression of OAT, BHMT, NLRP1, ECHS1 and PRDX1 was verified by Western blot analysis. The data are expressed as the mean ± SD. Compared to HBV (−) mouse:* *P* < 0.05, ** *P* < 0.01, *** *P* < 0.001

To verify the results of differentially transcribed genes, we tested the expression of *Nlrp1, Tcof1, Idh1, Nxn, Blvrb, GSTP2* and *Bscl2* in the liver of the HBV (+) mouse. These genes were related to mitochondrial fatty acid beta-oxidation, oxidative stress, and inflammation. The results were consistent with the RNA-Seq analysis; the RT-qPCR showed increased expression of *Nlrp1, Tcof1, Blvrb, Bscl2, NXN* and *GSTP2* and decreased expression of Idh1 in the liver of the HBV (+) mouse (Fig. 5a).

To verify the differentially expressed proteins that were identified by MALDI-TOF/TOF, a western blot was performed to assess the expression status of five identified proteins: OAT, BHMT, NLRP1, ECHS1 and PRDX1, which are related to mitochondrial fatty acid beta-oxidation, oxidative stress, inflammation, and amino acid metabolism. Consistent with the results of proteomics, the results of western blot analysis showed increased expression of BHMT, NLRP1, ECHS1, PRDX1, and Ubiquitin modified PRDX1 in the HBV (+) mouse, compared with those of the HBV (−) mouse. Additionally, the decreased expression of OAT was verified (Fig. 5b). Furthermore, the WB results were also consistent with the data of RT-qPCR (Fig. 5b).

Hepatic stellate cells play a key role in the development of liver fibrosis, and their activation is a major event in the process of liver fibrosis. Activated HSCs are the key source of the extracellular matrix and cytokines, which could further regulate the process of fibrosis. To further assess the changes of differentially expressed proteins in LX2 cells (HSC cell line), we first examined the expression of well-known fibrotic biomarkers, such as *α-SMA, TGF-β, Collagen I* and *Collagen III* in LX2 cells. As shown in Additional file 1: Fig. S3, these genes were up-regulated. These results indicated that LX2 cells were activated and produced extracellular matrix. Then, we assessed the expression of *Tcof1, Prdx1, Dag1b* and *Blvrb* in LX2 cells. Among these genes, *Prdx1* and *Blvrb* are related to the metabolism of reactive oxygen species. *Dag1b*is involved in the ECM-receptor interaction pathway. *Tcof1* is a randomly selected overlapped gene. The results showed that the expression of these genes significantly changed with the activated stellate cells (Additional file 1: Fig. S3). These results suggest that the differential expression of these genes may relate to the activation of HSCs.

## Discussion

Chronic hepatitis B virus infection remains the major cause of liver fibrosis and poses a serious threat to human beings. For this reason, many omics approaches have been applied to elucidate the mechanisms of HBV-associated liver fibrosis. However, the lack of reliable in vivo infection systems and, most importantly, convenient small animal models has restricted the study of HBV-related fibrosis mechanisms. In the present study, we established a mouse model with a persistent HBV infection and liver fibrosis. Compared with the HBV (−) mouse, the changes in parameters associated with fibrosis showed a significant difference at the RNA (Fig. 1a–d) and protein (Fig. 1e–g) levels. More importantly, extensive collagen deposition with an increasing trend was clearly displayed in the liver tissue during the 6-month period. Fibrous expansion was demonstrated around portal areas with marked bridging in the liver of the mouse model (Fig. 1h). These results demonstrated that the mouse model shows significant liver fibrosis after the AAV-HBV injection. Thus, this model is the closest to human HBV-induced fibrosis and the most suitable for the study of the mechanism of HBV-related liver fibrosis.

Liver fibrosis is a dynamic process. The changes in factors or pathways may be aggravated by cumulative effects, and others may be overshadowed as the disease progresses. In the present study, to further elucidate the mechanism of HBV-related fibrosis, continuous changes at the RNA and protein level were analyzed through RNA-Seq and 2DE-MALDI-TOF/TOF. Our results demonstrated that the protein and RNA profiling in the HBV (+) mouse was significantly different from that in the HBV (−) mouse. In total, 173 proteins and 6638 genes were differentially expressed, and 28 overlapped proteins were identified by comprehensive analysis of proteomic and transcriptomic data. GO, KEGG and DAVID analysis indicated that the differentially expressed proteins are primarily involved in oxidative stress (Figs. 2b, 3c, e and 4a, b), and many signal pathways were changed during HBV-related fibrosis, such as the NOD-like receptor signaling pathway, apoptosis, Toll-like receptor signaling pathway (Fig. 2c), metabolism-cytochrome P450, peroxisome and adipocytokine signaling pathway (Fig. 3d and f). More importantly, among these pathways, the RIG-I like receptor signaling pathway was commonly observed between the two omics analysis.

**Fig. 6:**
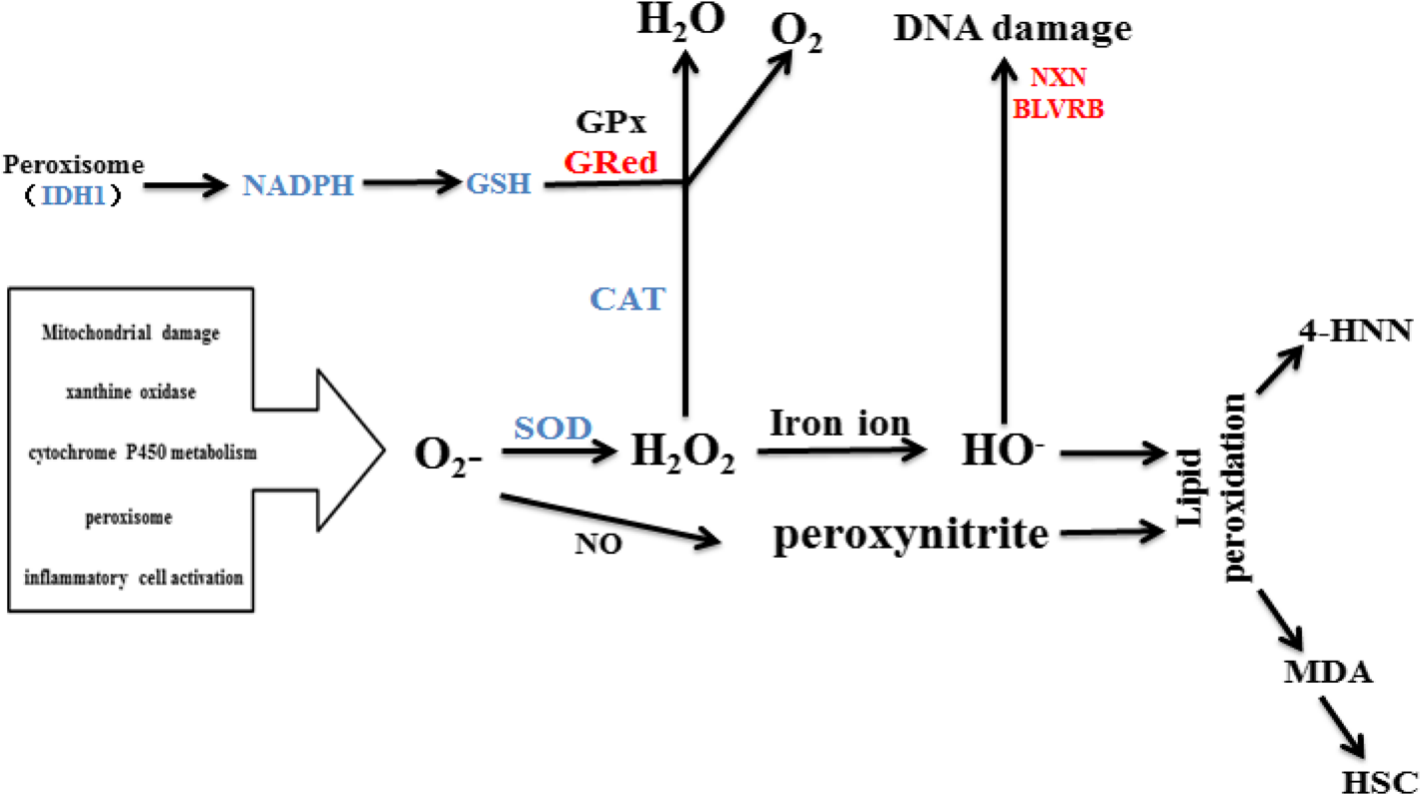
General pathways of ROS generation and antioxidant mechanisms. GPx: glutathione peroxidase, GRed: glutathione reductase, CAT: catalase, 4-HNN: 4-hydroxynonenal, MDA: malondialdehyde

Liver fibrosis caused by persistent HBV infection is the result of long-term complex interactions between the virus and host. The virus can hijack the host’s metabolism to obtain energy and complete proliferation [25]. This process may result in mitochondrial respiratory chain dysfunction and the overproduction of oxidative stress (OS), which reflects an imbalance between pro-oxidant/antioxidant redox. Persistent oxidative stress is a marker of chronic HBV infection, which is associated with many liver diseases, such as liver fibrosis, cirrhosis, and hepatocellular carcinoma [26]. The general pathways of ROS generation and antioxidant mechanisms are shown in Fig. 6. The major sources of intracellular oxidative stress include mitochondrial damage, xanthine oxidase, cytochrome P450 metabolism, peroxisome and inflammatory cell activation. There are several different enzymatic antioxidant defenses synthesized by the liver, including glutathione peroxidase (GPx)/reductase (GRed), superoxide dismutase (SOD), glutathione transferase, catalase (CAT), paraoxonase 1 and peroxiredoxin (Prx).

In the present study, the proteins that belong to the enzymatic antioxidant defenses showed significantly different expression (Fig. 4a, Table 1 and Additional file 1: Table S1). CAT and Idh1, which are located in the peroxisome, were down-regulated. Previous studies showed that CAT not only alleviates oxidative stress but also reduces HBx protein levels [27]. Idh1 protects murine hepatocytes from endotoxin-induced oxidative stress by regulating the intracellular NADP (+)/NADPH ratio [28]; in addition, it could affect the yield of GSH. Superoxide dismutase 1 (SOD1), which is located in the peroxisome and mitochondrion, could combine with copper and zinc ions and convert the harmful O_2_^−^ into O_2_ and H_2_O_2_. In contrast, BLVRB, NXN, and PRDX1, which are located in the nucleus and involved in antioxidants, were shown to be up-regulated. BLVRB is a component of antioxidant stress that has a cell protective function [29]. Nucleoredoxin (NXN) is a member of the antioxidant thioredoxin enzyme family that retains a pool of inactive disheveled (DVL) through a redox-sensitive interaction protein [30]. Peroxiredoxin 1 (PRDX1) belongs to the PRDX family, which is composed of thiol-specific antioxidant enzymes that reduce H_2_O_2_ and peroxynitrite [31, 32], and is involved in the mitigation of oxidative damage [33]. Moreover, Glutathione GSTP2, which is related to glutathione metabolism, was up-regulated during the entire 6-month period.

Based on our study, we propose a hypothesis of the mechanism of fibrosis. Firstly, in the process of HBV-induced liver fibrosis, some metabolic functions of the liver are disordered, such as lipid metabolism and energy metabolism. Then, the redox balance of pro/antioxidants is disrupted. Cytochrome P450 metabolism is abnormally activated, which results in increased intracellular oxidative stress. Additionally, the proteins localized in peroxisomes are down-regulated, which may lead to a decrease in the antioxidant capacity of the cells. These may be the major cause of increased intracellular oxidative stress. Secondly, due to the down-regulation of SOD1, the O_2_^−^ cannot be completely catalyzed to H_2_O_2_. O_2_^−^ rapidly reacts with NO to form the potent oxidant peroxynitrite, which in turn reacts with a number of important biological molecules, including lipids, glutathione (GSH), nucleic acids, and proteins, leading to cellular death. Thirdly, due to the down-regulation of Idh1, the synthesis of NADPH and GSH is reduced; however, the consumption of GSH is increased as a result of the up-regulation of GRed. These two changes eventually lead to the depletion of GSH in the process of H_2_O_2_ metabolism, resulting in excessive accumulation of H_2_O_2_. Furthermore, the down-regulation of CAT increases the accumulation of intracellular H_2_O_2_ as well as ROS. Excessive H_2_O_2_ can react with iron ions to form OH-, which further leads to DNA damage and lipid peroxidation. Fourthly, several antioxidant proteins localized in the nucleus, such as BLVRB, NXN, PRDX1, appeared to be up-regulated, which is beneficial for the protection of nuclear DNA from OH- damage. However, mitochondrial DNA may suffer more injury. In conclusion, HBV may decrease the intracellular antioxidant capacity, which results in the accumulation of intracellular oxidative stress, lipid peroxidation and DNA damage, and finally the formation of liver fibrosis.

One of the major causes of hepatitis is that the immune system attacks infected liver cells. The activation of RIG-I-like receptor signaling pathways, diverse viral RNAs, and triggers of the immune responses are changed during the process of liver fibrosis. Previous studies have implied that activation of the RIG-I pathway is effective in inhibiting HBV replication [34]. As observed in the present study (Additional file 1: Table S1), Ankrd17 (up-regulated), Traf3, and Nfkb1 (down-regulated) cluster into an innate immune response and are involved in this pathway. Ankrd17 is a positive regulator of the RLR signaling pathway. Overexpression of ankrd17 enhances RLR-mediated activation of IRF-3 and NF-κB and up-regulates the transcription of IFN-β [35, 36]*.* The expression level of Traf3 is decreased in chronic HBV-infected patients, and this protein can interact with the HBX protein [37]. Nfkb1 can regulate human NK cell maturation and effector functions [38, 39]. These results suggest that in the course of HBV infection, the host itself regulates the activity of the RIG pathway through Ankrd17 to enhance the immune response, but the HBx protein may inhibit this effect. Specific mechanisms need to be further studied.

In conclusion, liver fibrosis induced by HBV persistent infection has been extensively investigated, but the molecular mechanisms require further investigation. In particular, there is a lack of long-term studies on the molecular mechanisms of hepatic fibrosis. In this study, we established a mouse model of persistent HBV infection with liver fibrosis and then continuously analysed the profiles of genes and proteins using RNA-Seq and 2DE-MALDI-TOF/TOF to investigate the long-term molecular mechanism of liver fibrosis. This study showed that HBV-related fibrosis mobilizes different biological processes, multi-targets and multi-pathways, including oxidative stress and oxidation-reduction processes, whereas the RIG-I-like receptor signaling pathway regulates the target genes. This study provides novel insights into HBV-associated liver fibrosis and reveals the significant roles of oxidative stress in liver fibrosis. Furthermore, CAT, BLVRB, NXN, PRDX1, and IDH1 may be candidates for the detection of liver fibrosis or therapeutic targets for the treatment of liver fibrosis.

## Conclusions

This study provides novel insights into the HBV-associated liver fibrosis and reveals the significant roles of oxidative stress in liver fibrosis. Furthermore, CAT, BLVRB, NXN, PRDX1, and IDH1 may be candidates for the detection of liver fibrosis or therapeutic targets for the treatment of liver fibrosis.

## Materials and methods

### Animal model

Normal male C57BL/6 mice (aged 6–8 weeks; Vitalriver, Beijing, China) were bred and maintained at the Laboratory Animal Facility of the Institute of Laboratory Animal Sciences, Chinese Academy of Medical Science, Beijing. Animal care and procedures were performed in accordance with the Guide for the Care and Use of Laboratory Animals, which was approved by the Institutional Animal Care and Use Committee at the Chinese Academy of Medical Science.

AAV8-HBV1.2 vector was diluted into phosphate-buffered saline (PBS), and then, 200 μl of rAAV8-HBV 1.2 vector [2 × 10^11^ vector genome equivalents (vg)] was injected into the mice via the tail vein. The control group was injected with the same volume of PBS. Serum and liver tissues were collected at 1, 3 and 6-months post injection, and were frozen in liquid nitrogen.

### Protein preparation

Liver protein extraction was performed according to the manufacturer’s protocol (FOCUS- Mammalian Proteome, G-Biosciences, USA). First, 200 mg of liver tissue was added to 1.0 ml FBS buffer. The suspension was sonicated with an ultrasonic probe to break down the cells and genomic DNA. Sonication was performed in the cold (ice cold bath). During sonication, care was taken to prevent heating. Sonication was also performed in bursts of 20–30 s, and then, the suspension was chilled between ultra-sonic bursts. The homogenate was centrifuged at 20,000 x *g* for 30 min at 20 °C to pellet the tissue debris. A pipette was used to transfer the clear extract supernatant into a clean tube without disturbing the pellet. Any residual cell debris was suspended in 1/4 the volume of FPS Buffer used in the previous step. The suspension was sonicated again for 30 s. Then, the homogenate was centrifuged at 20,000 x *g* for 30 min at 20 °C to pellet the tissue debris. The extract was collected and pooled with the first extract supernatant. The total protein extract was stored at −80 °C for proteomic analysis and western blot.

### Two-dimensional gel electrophoresis and MALDI-TOF/TOF

The liver tissue protein concentration was measured according to the manufacturer’s protocol. First, 300 μg of liver tissue protein was collected from HBV (+) mice and HBV (−) mice at 1, 3 and 6-month post injection. These samples were analyzed by two-dimensional gel electrophoresis in triplicate. IPG strips (24 cm, nonlinear pH gradient range 4–10, GE Healthcare) were passively rehydrated with 300 μg liver tissue proteins. First-dimensional separation or isoelectric focusing (IEF) was performed in the EttanIPGphor III System (GE Healthcare) at 20 °C, using a stepwise mode to reach 97,250 vh with a limited current of 50 mA/strip. The strips were equilibrated for 15 min in an equilibration buffer containing 6 M urea, 112 mM Tris-base, 30% glycerol, 130 mM DTT, 4% SDS and 0.002% bromophenol blue after completion of the IFE. They were then incubated in another similar buffer that replaced DTT with 135 mM iodoacetamide for another 15 min. The second-dimensional separation was performed in 12% polyacrylamide gel using a SE260 Mini-Vertical Electrophoresis Unit (GE Healthcare) at 4 W for approximately 20 h. Finally, silver staining was performed to analyze the resolved protein spots.

Stained gels were scanned using an image scanner (Epson) in transmission mode. Analysis of the gels was accomplished using the Image Master™ 2D Platinum 7.0 (GE), including background subtraction, spot detection and the establishment of a reference gel. In-gel digestion of 2-D PAGE resolved proteins was performed using the following steps: interesting protein spots were excised from the 2-D gels and washed twice in MilliQ water. The gel pieces were destained in 15 mM potassium ferricyanide and 50 mM sodium thiosulfate and then rinsed twice in MilliQ water and once in 100 mM ammonium bicarbonate. Next, they were dehydrated in acetonitrile until the gel pieces turned opaque and white and were then dried in a centrifugal vaporizer. After the gel pieces were reduced, reducing buffer containing 10 mM DTT/100 mM Ambic was added to the EP tube containing the gel pieces and incubated in a water bath at 56 °C for 45 min. The solution was removed, and 55 mM iodoacetamide/100 mM Ambic was added immediately. The EP tubes were incubated at room temperature for 30 min in the dark. The gel pieces were incubated with 100 mM Ambic and ACN/Amibc (*v*/v 1:1) for 15 min. After incubation with 100% ACN for 5 min, then gel pieces were dried in a centrifugal vaporizer. An in-gel digestion that consisted of 50 mM ammonium bicarbonate, pH 8.0, and contained 100 ng of trypsin was added to suspend the gel pieces. The gel pieces then were incubated for 2 h at 4 °C and then overnight at 37 °C.

The supernatants that contained the peptide mixtures were removed and placed into a new EP tube, and the gel pieces were re-extracted with acetonitrile. The two fractions were collected in the same EP tube.

### MALDI MS analysis

Three microliters of peptide sample was thoroughly mixed with one microliter of R-cyano-4-hydroxycynnamic acid and the matrix (10 mg/mL in 0.2% trifluoroacetic acid (TFA) in 70% acetonitrile). The mixture was applied to the metallic sample plate and air-dried. Mass calibration was performed using the standard mixture provided by the manufacturer. Mass signals were then used to search through the database using the MASCOT peptide fingerprinting search program (version 2.3.02, Matrix Sciences, UK). The MS/MS spectra were searched against the databases of the UnProt mouse liver. Protein and peptide filtering and grouping options, which were embedded in the automation client program, were used to sort and filter the data. Enzyme specificity was set to trypsin/P, and a maximum of two missed cleavages was allowed. The initial maximal allowed mass tolerances were set at 50 ppm for precursor masses and at 0.5 Da for Fragment Mass Tolerance. The MS instrument model is an Ultraflex III MALDI-TOF/TOF, and the manufacturer is Bruker.

### RNA extraction, cDNA synthesis and sequencing

The total RNA of each sample was extracted using a RNeasy Mini Kit (Qiagen) and quantified with an Agilent 2100 Bioanalyzer (Agilent Technologies, Palo Alto, CA, USA), a NanoDrop spectrophotometer (Thermo Fisher Scientific Inc.), and 1% agarose gel. First, 1 μg of total RNA with a RIN value above 7 was used for following the library preparation. Next, generation sequencing library preparations were constructed according to the manufacturer’s protocol (NEBNext® Ultra™ RNA Library Prep Kit for Illumina®).

NEBNextPoly(A) mRNA Magnetic Isolation Module (NEB) and Ribo-Zero™ rRNA removal Kit (Illumina) were used to isolate the poly (A) mRNA. The mRNA fragmentation and priming were performed using NEB Next Random Primers and NEB Next First Strand Synthesis Reaction Buffer. ProtoScript II Reverse Transcriptase and Second Strand Synthesis Enzyme Mix were used to synthesize the first strand cDNA and second-strand cDNA, respectively. Double-stranded cDNA was purified by AxyPrep Mag PCR Clean-up (Axygen) and then treated with End Prep Enzyme Mix to repair both ends and add a dA-tailing in one reaction, followed by a T-A ligation to add adaptors to both ends. Fragments of approximately 360 bp (with the approximate insert size of 300 bp) were recovered using AxyPrep Mag PCR Clean-up (Axygen).

Each sample was then amplified by PCR for 11 cycles using P5 and P7 primers, with both primers carrying sequences that can anneal with flow cell to perform bridge PCR and P7 primer carrying a six-base index allowing for multiplexing. AxyPrep Mag PCR Clean-up (Axygen) was used to clean up the PCR products. Then, the PCR products were validated and quantified using the Agilent 2100 Bioanalyzer (Agilent Technologies, Palo Alto, CA, USA) and a Qubit 2.0 Fluorometer (Invitrogen, Carlsbad, CA, USA), respectively. The cDNA libraries with different indices were multiplexed and loaded on an Illumina HiSeq instrument according to the manufacturer’s instructions (Illumina, San Diego, CA, USA). Sequencing was carried out using a 2 × 150 bp paired-end (PE) configuration. Then, base calling and image analysis were performed by the HiSeq Control Software (HCS) + OLB + GAPipeline-1.6 (Illumina) on the HiSeq instrument. The sequences were processed and analyzed by GENEWIZ.

### Mapping and expression analysis

To remove technical sequences, including primers of polymerase chain reaction (PCR), adapters or fragments, and quality of bases lower than 20, the pass filter data in the fastq format was processed by Trimmomatic (v0.30) to ensure that clean data of the highest quality were obtained. Gene model annotation files of *Mus musculus* and reference genome sequences were downloaded from a genome website, such as NCBI, UCSC, ENSEMBL. Then, the genome sequence was indexed by Hisat2 (v2.0.1). Finally, clean data were aligned to reference genome via software Hisat2 (v2.0.1). Firstly, fasta format transcripts were converted from known gff annotation file and indexed properly. Then, the HTSeq (v0.6.1) estimated the gene and isoform expression levels from the pair-end of the clean data with the file as a reference gene file.

### Differential expression analysis

The DESeqBioconductor package, a model based on the negative binomial distribution, was used to analyze the differentially expressed genes. *P*-value of genes was set <0.05 to detect differential expressed genes after they were adjusted by Benjamini and Hochberg’s approach for controlling the false discovery rate (FDR).

### Bioinformatics analysis

GO-Term Finder was used to identify Gene Ontology (GO) terms. Enriched genes were annotated with a significant *p*-value of less than 0.05. KEGG (Kyoto Encyclopedia of Genes and Genomes) is a collection of databases dealing with biological pathways, genomes, drugs, chemical substances, and diseases (http://en.wikipedia.org/wiki/KEGG). In-house scripts were used to enrich significant differentially expressed genes and proteins in the KEGG pathway. STRING (version 10.0, http://string-db.org/) and Cytoscape software (v3.4.0) were used to analyze the protein-protein interaction (PPI) network. DAVID (v6.7, http:// david.ncifcrf.gov/ ) was used to perform a cluster analysis of the overlapped proteins.

### Quantitative RT-PCR (RT-qPCR)

First, 1 μg of the total RNA (described above for RNA-Seq) was reverse transcribed using the First Strand cDNA Synthesis kit (Toyobo, OSAKA, Japan) with the oligod(T)18 primer. Quantitative PCR was performed in triplicate in 96-well optical reaction plates using a Bio-Rad CFX96™ Real Time System and SYBR Green I PCR mix (Roche Diagnostics, Indianapolis, IN). PCR was performed with the following cycling conditions: an initial denaturation at 95 °C for 10 min followed by 40 cycles of 95 °C for 15 s and 55 °C for 30 s. The 2^-ΔΔC(t)^ method was used to calculate and determine the fold change. The primer sequences are shown in Additional file 1: Table S2.

### Elisa

The levels of TGF-β1 and TIMP1 in the serum samples and hydroxyproline in the liver tissue were determined using commercially available ELISA kits (R&D Systems, Minneapolis, MN).

### Immunohistochemistry

Immunohistochemistry was performed using the liver tissue, which was analyzed by proteomics and RNA-seq analyses. The liver sections were examined by light microscopy after Masson’s trichrome staining. The Sirus Red staining of liver sections was observed by polarizing microscopy.

### Western blot

20 μg of liver tissue proteins was separated by 12% SDS-polyacrylamide gel electrophoresis. The separated proteins were blotted onto PVDF membrane. Then, the membrane was washed with TBST and incubated in blocking buffer containing 5% BSA in TBST for 2 h at room temperature. The PVDF membrane was washed with TBST and finally incubated overnight at 4 °C with primary antibodies diluted in TBST, including rabbit anti-mouse Oat, Bhmt, Nlrp1b, Prdx1, Echs1 (Abcom), and β-Actin (Sigma). Next, the PVDF membrane was incubated with goat anti-rabbit secondary antibody (Cell Signaling Technology, Danvers, MA, USA) for 1 h at room temperature. Finally, the blot was scanned by infrared rays with the Odyssey Infrared Imager (LI-COR, Lincoln, NB, USA).

### Statistical analysis

The data are expressed as the mean ± SD. Statistical analysis was performed using one-way analysis of variance (ANOVA, GraphPad Prism 5) to determine statistically significant differences between groups. *P* < 0.05 was considered statistically significant.

## Additional file

Additional file 1: Figure S1. Protein-Protein Interaction was analyzed by STRING and Sytoscape software. **Figure S2.** Protein-Protein Interaction of the up-regulated and down-regulated genes was analyzed by STRING and Sytoscape software. **Figure S3.** The expression of representative genes in LX2 cells. **Table S1.** Functional annotation clustering of overlapping genes. **Table S2.** Gene specific primers used in real-time PCR conformation experiments. (DOCX 10032 kb)

## Abbreviations

4-HNN: 4-hydroxynonenal
AAV: Adeno-associated virus
Ankrd17: Ankyrin Repeat Domain 17
BHMT: Betaine--Homocysteine S-Methyltransferase
BLVRB: Biliverdin Reductase B
Bscl2: Bernardinelli-Seip congenital lipodystrophy type 2 protein
CAT: Catalase
Dbi: Acyl-CoA Binding ProteinDag1b:Dystroglycan 1
DEG: Differentially expressed genes
ECHS1: Enoyl-CoA Hydratase, Short Chain 1
ECM: Extracellular matrix
FDR: False discovery rate
GPx: Glutathione peroxidase
GRed: Glutathione reductase
GSTP2: Glutathione S-transferase P 2
HBV: Human hepatitis B virus
HCC: Hepatocellular carcinoma
HSC: Hepatic stellate cell
Idh1: Isocitrate Dehydrogenase (NADP(+)) 1
IEF: Isoelectric focusing
KEGG: Kyoto Encyclopedia of Genes and Genomes
MDA: Malondialdehyde
Nfkb1: Nuclear Factor Kappa B Subunit 1
NLRP1: NLR Family Pyrin Domain Containing 1
NXN: Nucleoredoxin
OAT: Ornithine Aminotransferase
PPI: protein-protein interaction
PRDX1: Peroxiredoxin 1
ROS: Reactive oxygen species
RPKM: The reads per kilobase of transcript per megabase library size
Tcof1: Treacle Ribosome Biogenesis Factor 1
TGF-β1: Transforming growth factor beta 1
TIMP1: Metalloproteinase inhibitor 1
Traf3: TNF Receptor Associated Factor 3
