# Supplementary material for: Proteomic and transcriptomic studies of HBV-associated liver fibrosis of an AAV-HBV-infected mouse model
Source: BMC Genomics. 2017 Aug 22;18:641. doi: 10.1186/s12864-017-3984-z (PMC5568174; doi:10.1186/s12864-017-3984-z)
Supplement: Additional file 1: Figure S1. — Protein-Protein Interaction was analyzed by STRING and Sytoscape software. Figure S2. Protein-Protein Interaction of the up-regulated and down-regulated genes was analyzed by STRING and Sytoscape software. Figure S3. The expression of representative genes in LX2 cells. Table S1. Functional annotation clustering of overlapping genes. Table S2. Gene specific primers used in real-time PCR conformation experiments. (DOCX 10032 kb) [file 12864_2017_3984_MOESM1_ESM.docx]

**Additional file 1**

**Table S1. Functional annotation clustering of overlapping genes**

| Annotation Cluster 1, Enrichment Score: 1.86 | | | | | | | | |
| --- | --- | --- | --- | --- | --- | --- | --- | --- |
|  | | RELATIVE GENE NAME | | Count | | P_Value | | Benjamini |
| GOTERM_BP_DIRECT | glutathione metabolic process | Gstp1,Gstp2,Idh1 | | 3 | | 2.40E-03 | | 5.10E-01 |
| KEGG_PATHWAY | Glutathione metabolism | Gstp1,Gstp2,Idh1 | | 3 | | 7.10E-03 | | 4.50E-01 |
| GOTERM_CC_DIRECT | extracellular exosome | Bhmt,Blvrb,Cat,Dbi,  Gstp1,Gstp2,Idh1 | | 7 | | 1.50E-01 | | 7.70E-01 |
| Annotation Cluster 2, Enrichment Score: 1.64 | | | | | | | | |
|  | | | RELATIVE GENE NAME | Count | P_Value | | Benjamini | |
| GOTERM_CC_DIRECT | cytosol | | Bhmt,Blvrb,Cat,Dbi,  Gstp1,Gstp2,Idh1,  Nfkb1,  Nxn,Uroc1 | 9 | 2.10E-03 | | 6.30E-02 | |
| UP_KEYWORDS | NADP | | Blvrb,Cat,Idh1 | 3 | 1.80E-02 | | 3.70E-01 | |
| UP_KEYWORDS | Oxidoreductase | | Blvrb,Cat,Idh1,Nxn | 4 | 3.90E-02 | | 4.90E-01 | |
| GOTERM_MF_DIRECT | oxidoreductase activity | | Blvrb,Cat,Idh1,Nxn | 4 | 5.40E-02 | | 9.40E-01 | |
| GOTERM_BP_DIRECT | oxidation-reduction process | | Blvrb,Cat,Idh1,Nxn | 4 | 7.80E-02 | | 9.70E-01 | |
| Annotation Cluster 3 Enrichment Score: 1.24 | | | | | | | | |
|  | | RELATIVE GENE NAME | | Count | P_Value | | Benjamini | |
| GOTERM_BP_DIRECT | innate immune response | Nlrp1b,Traf3,Ankrd17,  Nfkb1 | | 4 | 2.10E-02 | | 7.90E-01 | |
| UP_KEYWORDS | Immunity | Nlrp1b,Traf3,Ankrd17, | | 3 | 8.20E-02 | | 6.10E-01 | |
| GOTERM_BP_DIRECT | immune system process | Nlrp1b,Traf3,Ankrd17, | | 3 | 1.10E-01 | | 9.80E-01 | |
| Annotation Cluster 4 Enrichment Score: 1.09 | | | | | | | | |
|  | | RELATIVE GENE NAME | | Count | P_Value | | Benjamini | |
| UP_KEYWORDS | Wnt signaling pathway | Ccar2,Nxn,Wnt2b | | 3 | 1.90E-02 | | 3.10E-01 | |
| GOTERM_BP_DIRECT | Wnt signaling pathway | Ccar2,Nxn,Wnt2b | | 3 | 4.00E-02 | | 8.60E-01 | |
| GOTERM_MF_DIRECT | protein binding | Arhgef1,Traf3,Ccar2,  Nfkb1,Nxn,Wnt2b | | 6 | 7.30E-01 | | 1.00E+00 | |

**Table S2. Gene specific primers used in real-time PCR conformation experiments.**

| Gene | Forward Primer (5’-3’) | Reverse Primer (5’-3’) |
| --- | --- | --- |
| Nlrp1b  (Mus musculus) | CATCCACATACTGCTCACTTC | TCTTCACACCACCATCACC |
| Echs1  (Mus musculus) | CAGTTGTTGGAAGTCAGG | TTGGGAATCAGCAGAGAG |
| Tcof1  (Mus musculus) | AGGAGGATGCCAAGAGAC | CTTCGCTGGATTCTGTAGG |
| Idh1  (Mus musculus) | CGAGGGAGGCTTCATCTG | TCTGCTTCTACCGTCTTACC |
| Nxn  (Mus musculus) | CCCAACACTTTACCAGCCCAAAC | AGCCAACAATCCAATTCAGCAAGG |
| Prdx1  (Mus musculus) | TTACCTGCCTGTTGGATACC | AGCCTCACATATACATCAAGTTC |
| Arhgap21  (Mus musculus) | ATTCTACCACATCATCCACTAC | TCGTCATCAGCCTCATCC |
| Bhmt  (Mus musculus) | AAGGACATAGCAACCAACTTTATC | TGGAATAGAAGCAGACAGAATAGC |
| Blvrb  (Mus musculus) | GAGACCAACCACTAACTG | TGTCCGTCATACTCATTG |
| Bscl2  (Mus musculus) | CGTCATCGTGCTCTTCAG | CCGTGGTGTGAGTTATCC |
| α-SMA | AGCCAGTCGCCATCAGGAAC | GGGAGCATCATCACCAGCAA |
| TGF-β  (Homo sapiens) | CAGCAGCAGCGACCAGAG | CAGTAGTAGGCGGCGTAGC |
| Collage I | GACATGTTCAGCTTTGTGGACCC | AGGGACCCTTAGGCCATTGTGTA |
| Dbi  (Homo sapiens) | CATTCCTCTCCTGAACTCTG | CCAACTAACTGCCTGAGC |
| Nlrp1b  (Homo sapiens) | TGTTGCTCAGGCTAATCTC | CAGAAGTCATCGTCAGTCC |
| Tcof1  (Homo sapiens) | AAGAAGAAGACAGCAGAGCAG | GTCGGTGGAGGTCAGAGG |
| Prdx1  (Homo sapiens) | AACACAAGACTTCAGATTCAGC | CCACAACGCCAACTCAGG |
| Nxn  (Homo sapiens) | AAGCAGAACTACAAAGATGAG | ATACAAGGCGACACAAGG |
| Dag1b  (Homo sapiens) | CAGGAACGATGGACTCAC | GCTCACTACTTCTGCTACC |
| Blvrb  (Homo sapiens) | ATAGGAGACCAGCCACTAAC | TGTCCGTCGTACTCATCG |
| β-Actin | CGTTGACATCCGTAAAGACC | TAGAGCCACCAATCCACACA |


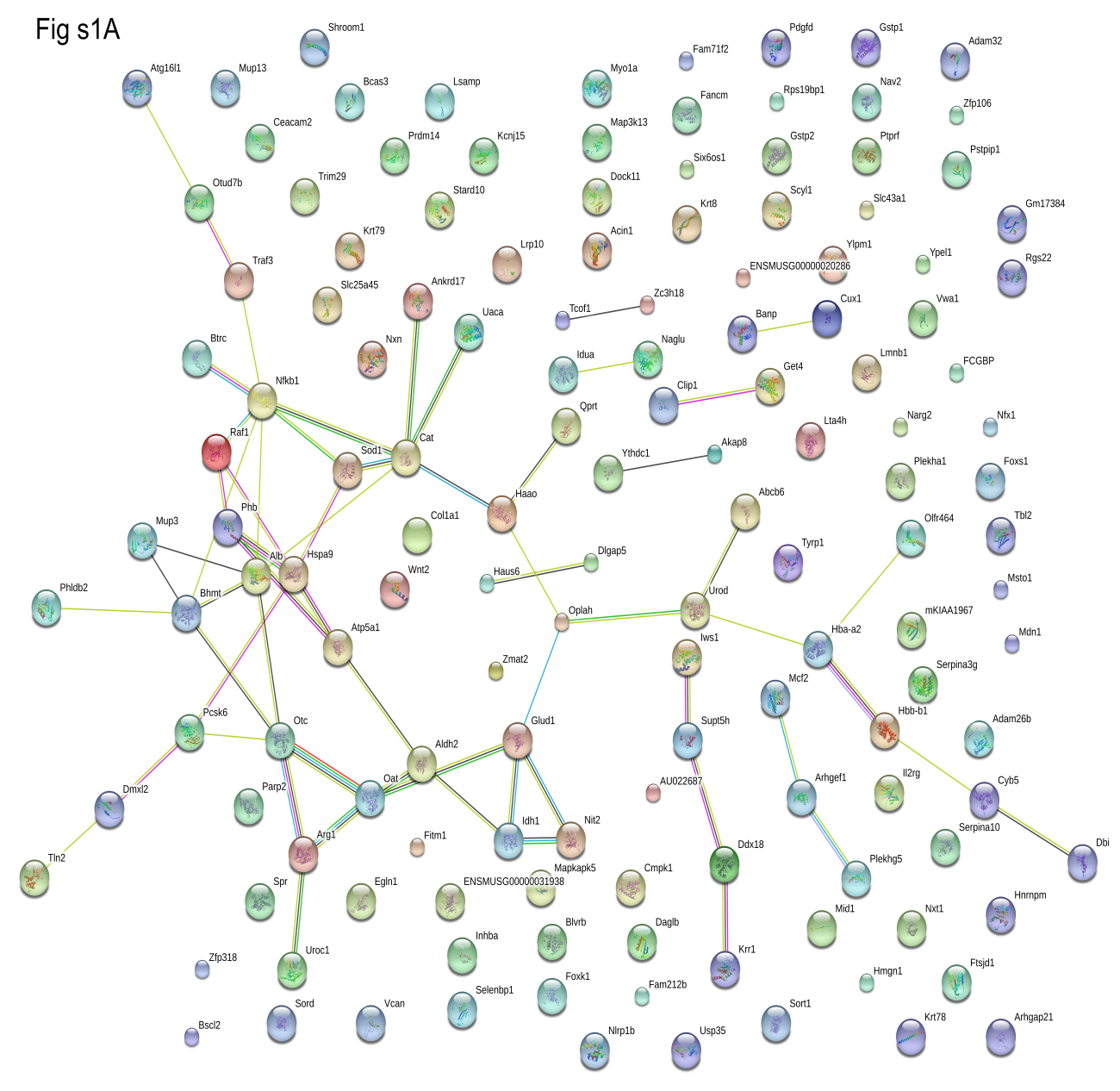


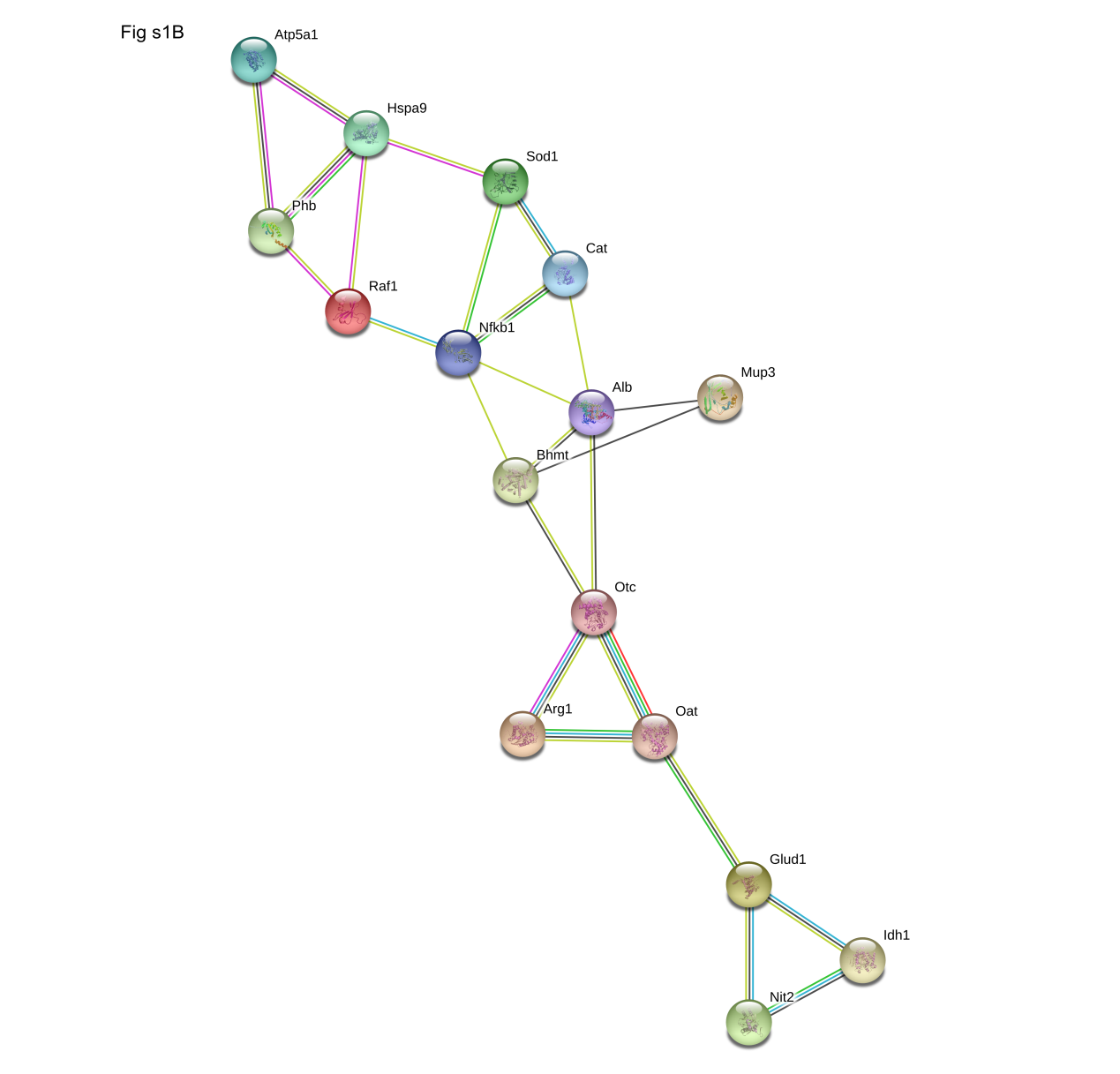


Figure S1. Protein-Protein Interaction was analyzed by STRING and Sytoscape software. The network of total differentially expressed proteins (A) and the key clusters (B) of the network were shown.


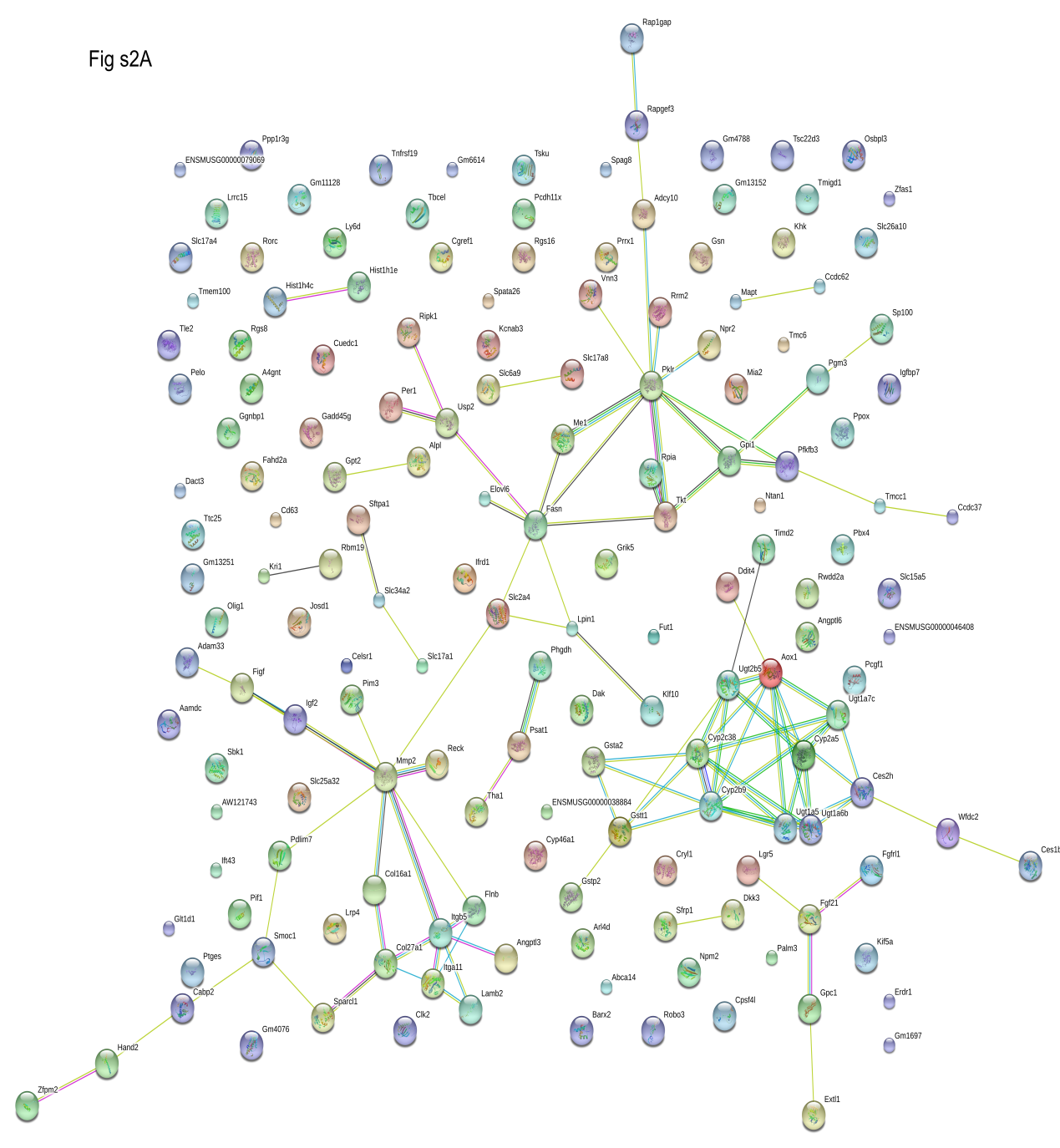


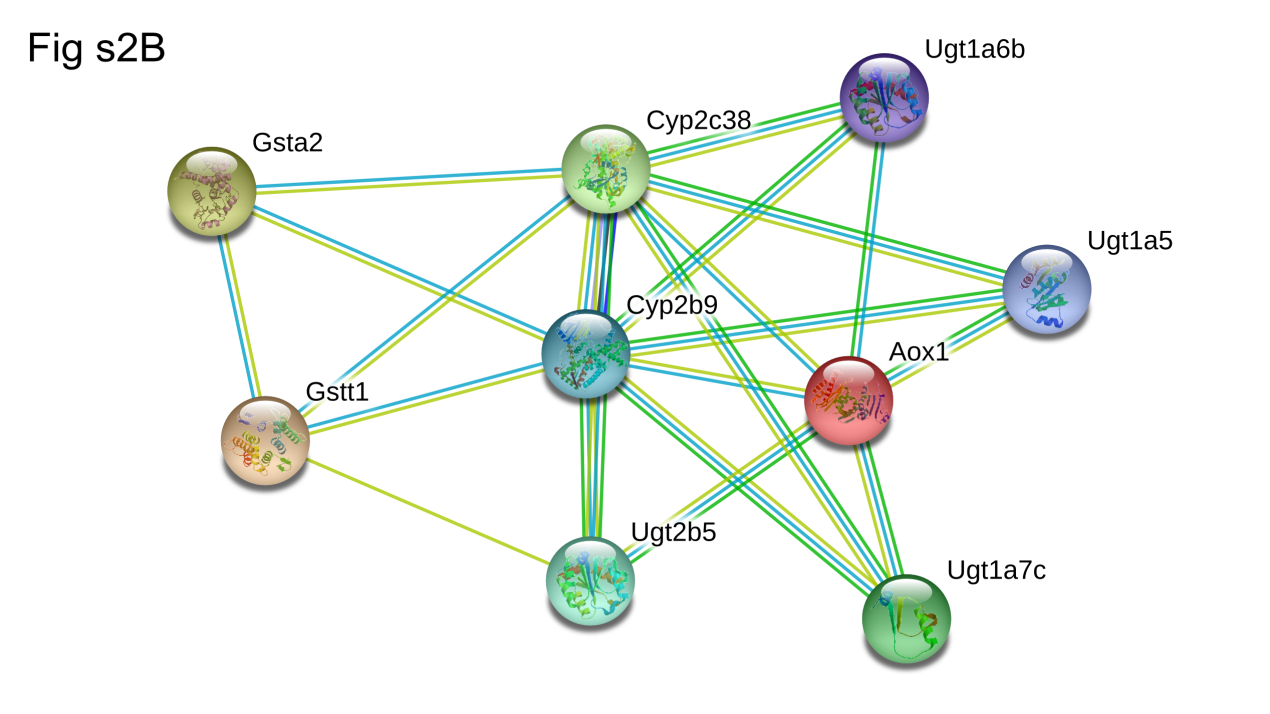


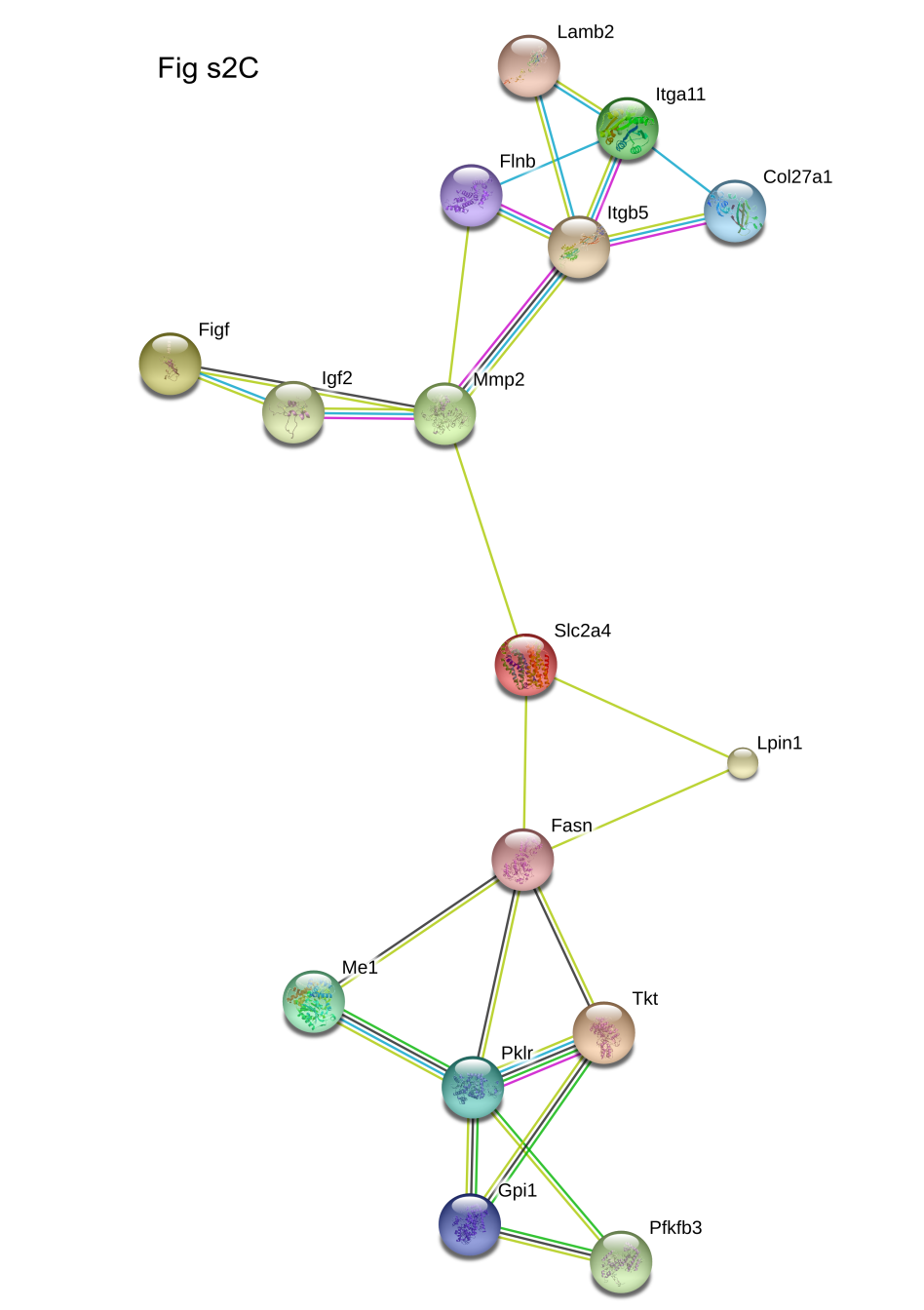


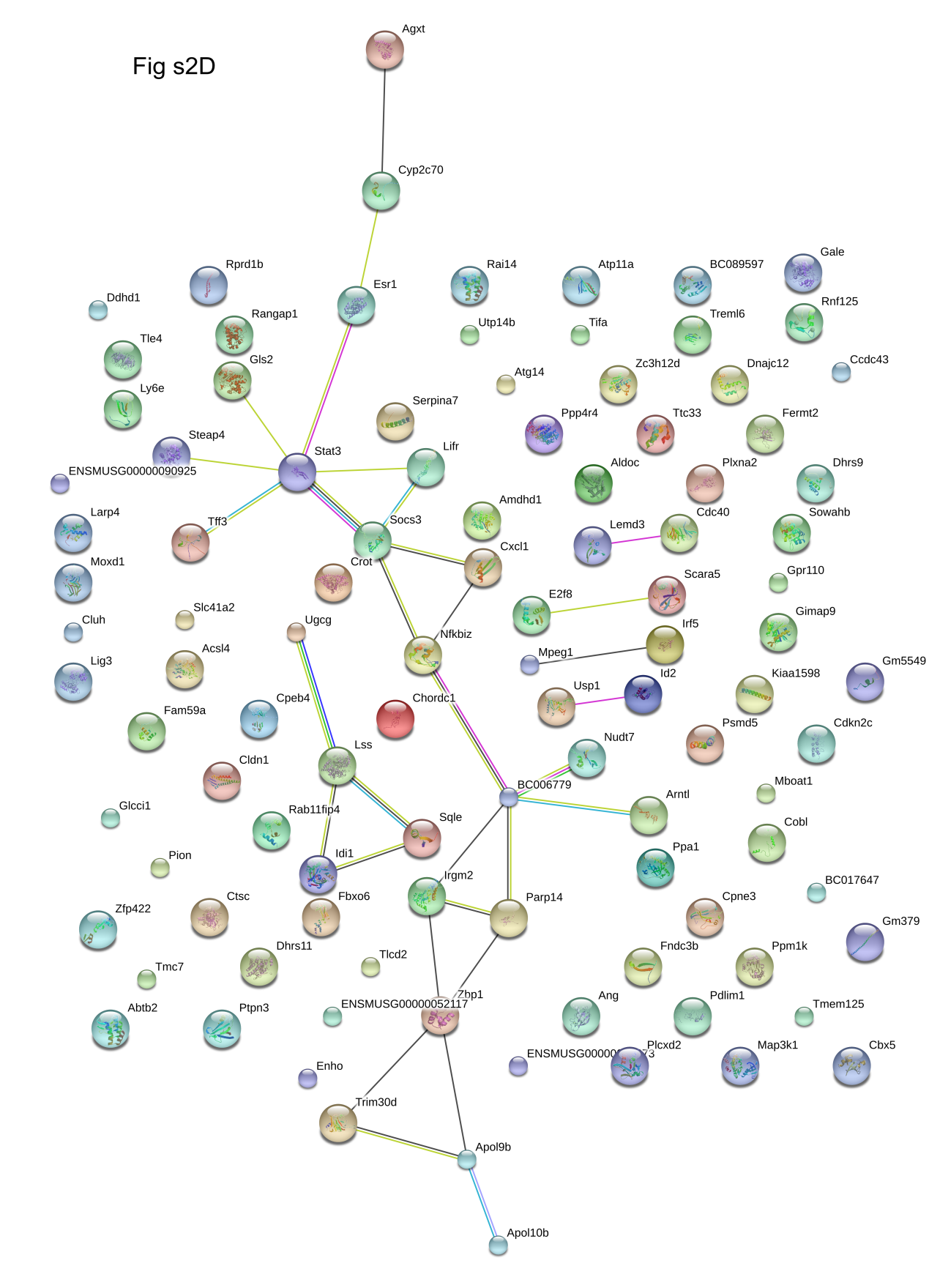


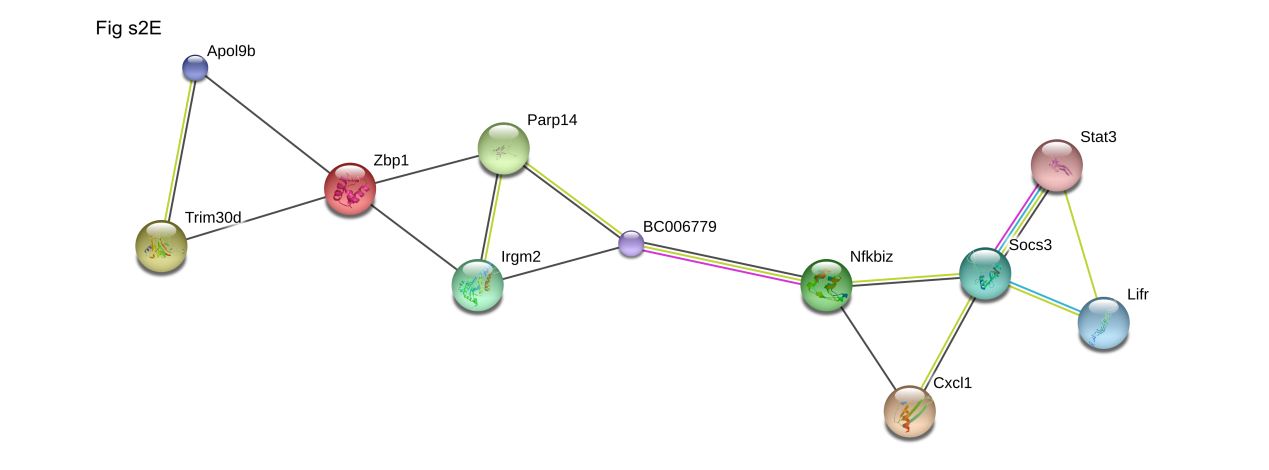


Figure S2. Protein-Protein Interaction of the up-regulated and down-regulated genes was analyzed by STRING and Sytoscape software. The whole interaction networks (A and D) and the key enrichment networks (B; C and E) were displayed.


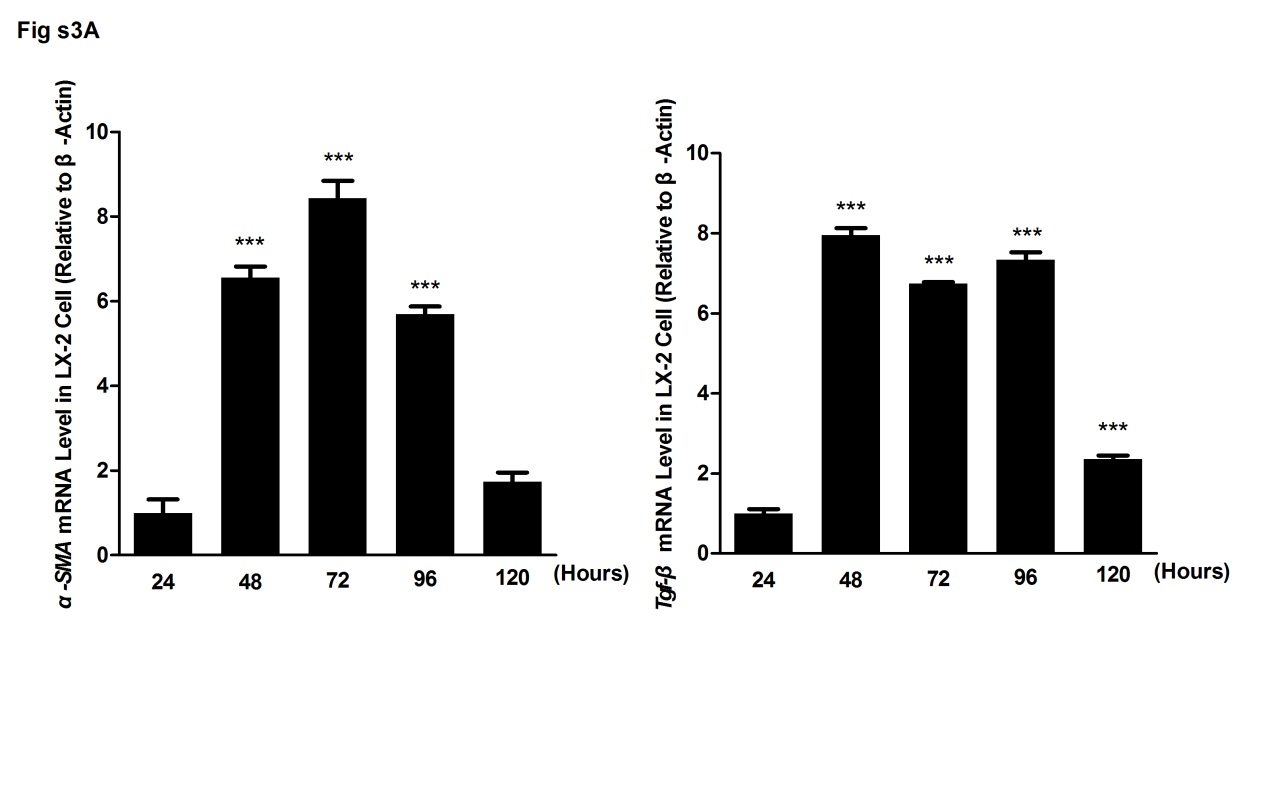


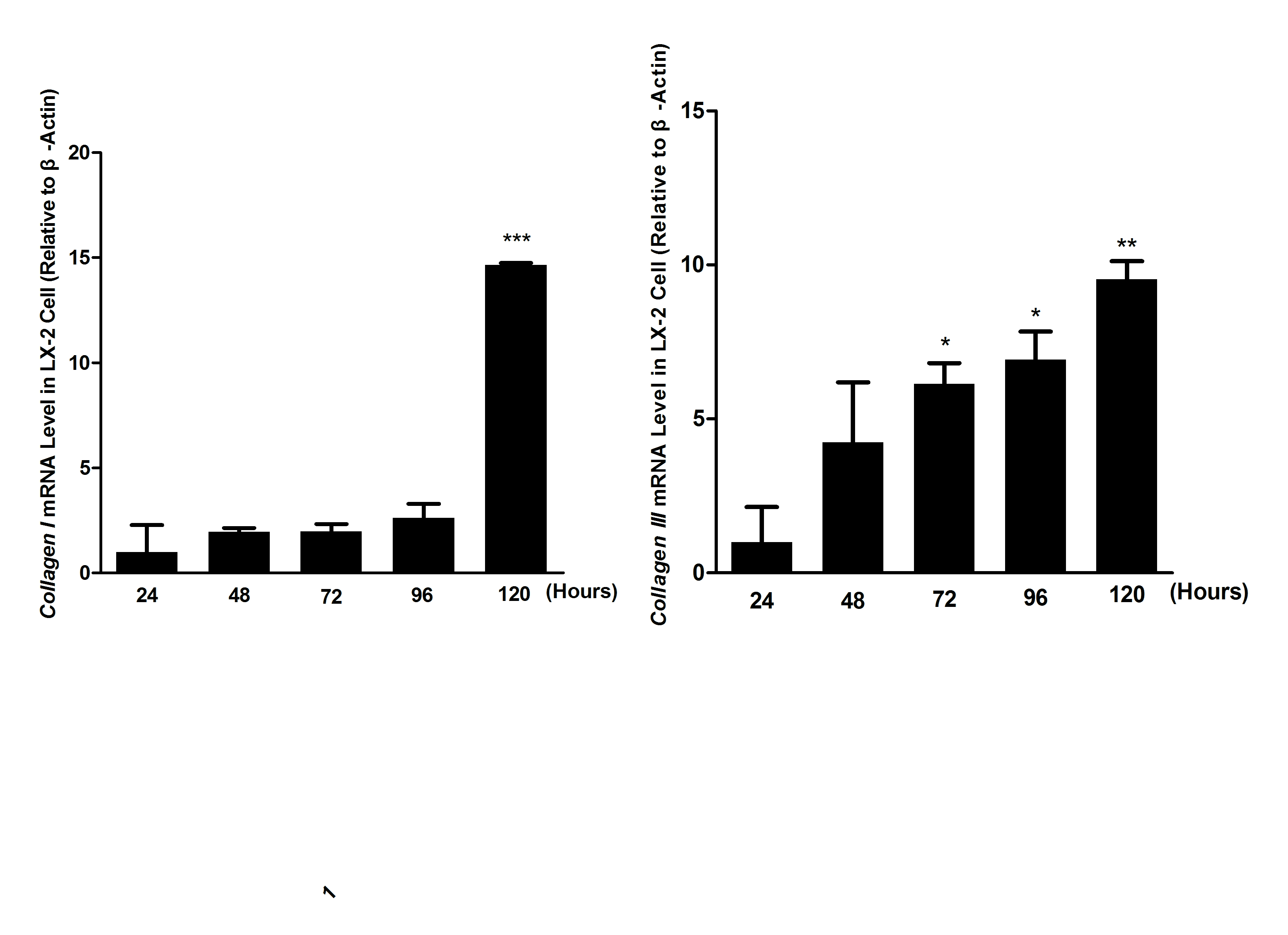


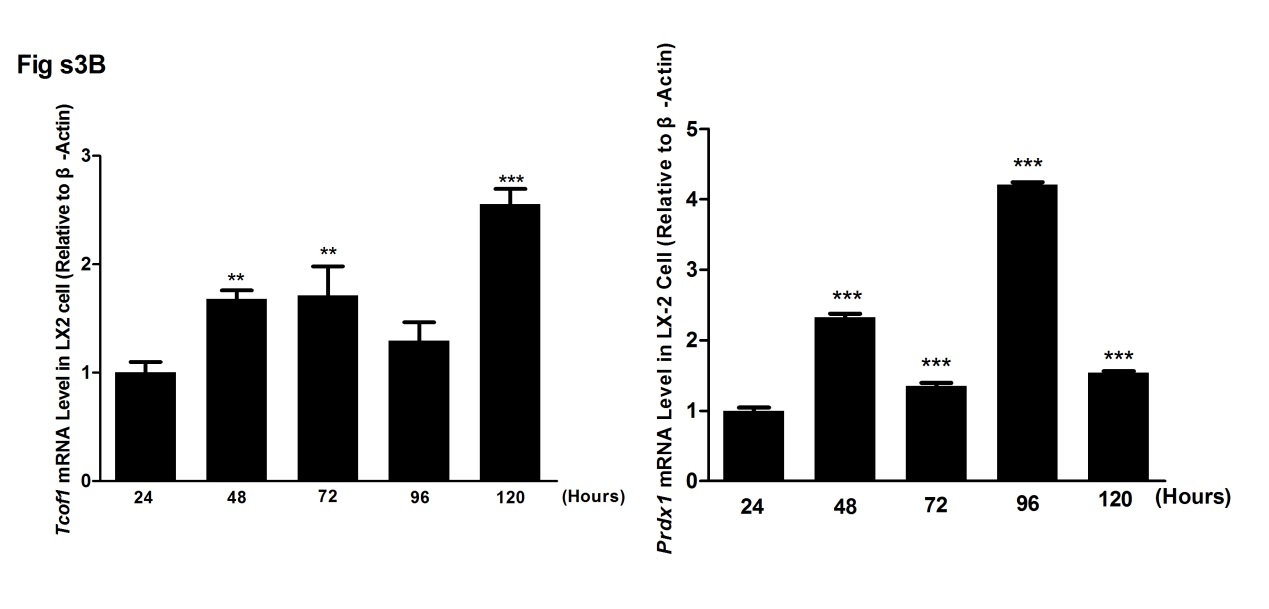

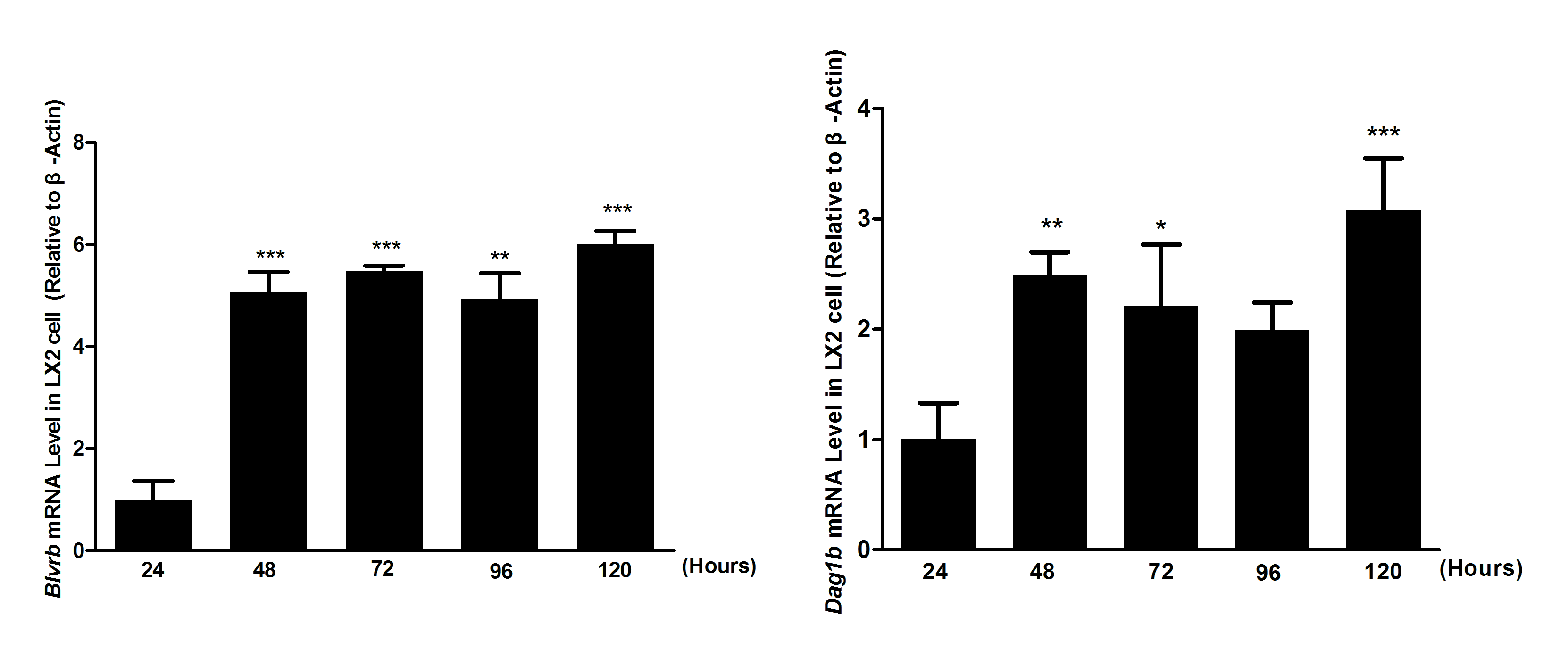


Figure S3. The expression of representative genes in LX2 cells. LX2 cells were cultured 120 Hours, and then the expression of four fibrosis related indexes (A) and four differentially expressed genes (B) were assessed in LX2 cells. Compared to 24 Hours:* P < 0.05, ** P < 0.01, ***P<0.001.
